# Genome-wide screen identifies novel genes required for *Borrelia burgdorferi* survival in its *Ixodes* tick vector

**DOI:** 10.1371/journal.ppat.1007644

**Published:** 2019-05-14

**Authors:** James P. Phelan, Aurelie Kern, Meghan E. Ramsey, Maureen E. Lundt, Bijaya Sharma, Tao Lin, Lihui Gao, Steven J. Norris, Jenny A. Hyde, Jon T. Skare, Linden T. Hu

**Affiliations:** 1 Department of Molecular Biology and Microbiology, Tufts University, Boston, Massachusetts, United States of America; 2 Department of Molecular Virology and Microbiology, Baylor College of Medicine, Houston, Texas, United States of America; 3 MD Anderson Cancer Center Thoracic & Cardiovascular Surgery, Houston, Texas, United States of America; 4 Department of Pathology and Laboratory Medicine, McGovern Medical School at UT Health, Houston, Texas, United States of America; 5 Department of Microbial Pathogenesis and Immunology, Texas A & M University Health Science Center, Bryan, Texas, United States of America; University of Montana, UNITED STATES

## Abstract

*Borrelia burgdorferi*, the causative agent of Lyme disease in humans, is maintained in a complex biphasic life cycle, which alternates between tick and vertebrate hosts. To successfully survive and complete its enzootic cycle, *B*. *burgdorferi* adapts to diverse hosts by regulating genes required for survival in specific environments. Here we describe the first ever use of transposon insertion sequencing (Tn-seq) to identify genes required for *B*. *burgdorferi* survival in its tick host. We found that insertions into 46 genes resulted in a complete loss of recovery of mutants from larval *Ixodes* ticks. Insertions in an additional 56 genes resulted in a >90% decrease in fitness. The screen identified both previously known and new genes important for larval tick survival. Almost half of the genes required for survival in the tick encode proteins of unknown function, while a significant portion (over 20%) encode membrane-associated proteins or lipoproteins. We validated the results of the screen for five Tn mutants by performing individual competition assays using mutant and complemented strains. To better understand the role of one of these genes in tick survival, we conducted mechanistic studies of *bb0017*, a gene previously shown to be required for resistance against oxidative stress. In this study we show that BB0017 affects the regulation of key borrelial virulence determinants. The application of Tn-seq to *in vivo* screening of *B*. *burgdorferi* in its natural vector is a powerful tool that can be used to address many different aspects of the host pathogen interaction.

## Author summary

*Borrelia burgdorferi*, the causative agent of Lyme disease, must adjust to environmental changes as it moves between its tick and vertebrate hosts. We performed a screen of a *B*. *burgdorferi* transposon library using massively parallel sequencing (Tn-seq) to identify fitness defects involved in survival in its tick host. This screen accurately identified genes known to cause decreased fitness for tick survival and identified new genes involved in *B*. *burgdorferi* survival in ticks. All of the genes tested individually confirmed the Tn-seq results. One of the genes identified encodes a protein whose function was previously unknown that appears to be involved in regulating expression of proteins known to be involved in environmental adaptation. Tn-seq is a powerful tool for understanding vector-pathogen interactions and may reveal new opportunities for interrupting the infectious cycle of vector-borne diseases.

## Introduction

Lyme disease is caused by the spirochete, *Borrelia burgdorferi*. In nature, *B*. *burgdorferi* is maintained in a cycle between mammalian or bird hosts and *Ixodes* ticks [1] Newly hatched ticks can acquire *B*. *burgdorferi* from infected animals during their larval feeding [1]. After molting to the nymphal stage, those infected ticks can transmit the pathogen to a new vertebrate host during their next blood meal [1]. The challenges posed by the vertebrate and tick environments are quite different. *B*. *burgdorferi* must adapt to changes in temperature, pH, nutrient availability and immune defense mechanisms between its vertebrate and arthropod hosts [2–6].

Previous studies have shown that *B*. *burgdorferi* adapts to its host environments through controlling the expression of proteins that aid in survival at specific points in its life cycle in its different hosts [7–9]. For example, proteins such as outer surface protein C (OspC), variable-major- protein (Vmp)-like sequence E (VlsE) and decorin binding protein A (DbpA) are expressed to differing amounts during particular time points in the mammalian and tick phases of the *B*. *burgdorfer*i life cycle [10–14]. The regulation of gene expression in *B*. *burgdorferi* is complex, often involving multiple layers of control [1,3,6].

Expression of proteins required during the mammalian phase involves two alternative sigma factors, RpoS and RpoN, the enhancer binding protein Rrp2, as well as the transcription factors BosR and BadR [15–26]. In addition to controlling virulence gene expression, BosR also controls expression of genes involved in resistance to reactive oxygen species and affects metal homeostasis, while BadR controls expression of many genes involved in metabolite uptake and utilization [17,27, 28]. Other regulators such as carbon storage regulatory protein A (CsrA) appear to exert their effects outside the RpoS/RpoN axis [29].

Much less is known about gene regulation and proteins critical for *B*. *burgdorferi* survival while in its tick host [6]. Histidine kinase 1 (Hk1) and response regulatory protein 1 (Rrp1) are highly expressed during the tick phase and appear to work together to regulate expression of genes involved in tick survival [30–32]. Rrp1 is a diguanylate cyclase required for the synthesis of cyclic diguanylate (c-di-GMP), an important second messenger signaling molecule in *B*. *burgdorferi* and other bacteria [32–35] The exact mechanisms by which Hk1 is activated and how Rrp1 is regulated are not known.

Proteins that have been shown to be important in survival in ticks include outer surface protein A (OspA), which binds to the tick mid gut protein TROSPA [6,36]. GuaA and GuaB, two enzymes that contribute to the purine salvage pathway, have also been shown to provide a fitness advantage in the tick host [37]. The glycerol utilization operon (*glpF*, *glpK*, *glpD*) encodes proteins that allow the bacterium to utilize glycerol as the carbohydrate source for glycolysis [33,38]. This operon is upregulated during all tick life cycle stages, and has been shown to be specifically involved with persistence and survival of the molt, but not early colonization [33,35,38]. Another protein shown to be essential for infection of the tick host is the manganese transporter BmtA. This transporter is required for *B*. *burgdorferi* to colonize and survive in ticks [39].

In this study, we describe the use of transposon insertion sequencing (Tn-seq) to identify genes that are critical for *B*. *burgdorferi* survival during infection of *Ixodes scapularis*, the tick vector most commonly associated with Lyme disease transmission in North America [1]. Tn-seq is a high- throughput approach that enables the quantification of the frequency of individual transposon (Tn) mutants in a population before and after a selective pressure [40]. Tn-seq has been widely used for *in vitro* assays of bacterial fitness [41–44]. It has also been used to perform *in vivo* studies in mice, although *in vivo* Tn-seq studies are often limited by tight bottlenecks causing stochastic loss of mutants unrelated to the Tn insertion [40–43]. This report represents the first use of a Tn insertional library combined with massively parallel sequencing to identify bacterial genes involved in colonization of an arachnid. Using Tn-seq, we were able to accurately identify a number of *B*. *burgdorferi* mutants with impaired fitness for survival in *Ixodes* ticks. The process is easily scalable though testing additional ticks, which reduces misidentification of mutants that are lost for reasons other than fitness. As opposed to mammalian studies, in which the number of animals is often limiting, we were able to readily screen very large numbers of larval ticks, thereby mitigating bottleneck issues. As part of our studies, we have identified a potential new regulator of *B*. *burgdorferi* gene expression, BB0017, which may contribute to expression of genes involved in tick and mammalian survival.

## Materials and methods

### Ethics statement

Mice were bred and maintained in the Tufts University Animal Facility. All experiments were performed following the guidelines of the American Veterinary Medical Association (AVMA) as well as the Guide for the Care and Use of Laboratory Animals of the National Institutes of Health. All procedures were performed with approval of the Tufts University Institutional Animal Care and Use Committee (IACUC, Protocol# B2015-159). Euthanasia was performed in accordance with guidelines provided by the AVMA and was approved by the Tufts University IACUC.

### Bacterial strains and growth conditions

*B*. *burgdorferi* strains were grown in Barbour-Stoenner-Kelley II (BSK-II) medium in sealed tubes at 32°C with 1% CO2. *Escherichia coli* strains (Top10) for plasmid preparation were grown on Lysogeny broth (LB) agar plates or in LB broth at 37oC. *E*. *coli* cultures contained either 50 μg/ml spectinomycin or 10 μg/ml gentamicin. The parental strain of the Tn library, the infectious *B*. *burgdorferi* strain 5A18NP1, was used as the wild-type strain in all studies and lacks two plasmids (lp56 and lp28-4) [45]. The following antibiotics were used for selection in cultures of *B*. *burgdorferi* when appropriate: kanamycin at 200 μg/ml, gentamicin at 40 μg/ml, and streptomycin at 50 μg/ml. Tn mutants were obtained from the arrayed *B*. *burgdorferi* library [45]. All individual Tn mutants used in this study were screened by polymerase chain reaction (PCR) at the locus of interest to confirm pure populations as previously described [42, 43]. In cases where mixed populations were identified (i.e. two PCR products indicating the presence of both a wild-type and Tn disrupted locus), the strain was plated for single colonies in semi-solid agarose overlays. Individual colonies were then selected and re-screened to confirm pure populations. All Tn mutants were subsequently plasmid- typed to identify the loss of any plasmids required for murine or tick infection [46, 47]. A description of all individual Tn mutants used in this study is available in Table 1. Other *B*. *burgdorferi* strains as well as plasmids used in this study are also described in Table 1.

**Table 1:** Strains and plasmids used in this study.

Strain/ Plasmid Name	Description	Missing plasmids*	Ref/ Source
**Strain Name**			
5A18NP1	Parental strain of Tn library		[49]
Tn::*bb0017*	Tn mutant with Tn insertion in the reverse orientation at position 16662 in *bb0017* (insertion ratio 0.85); kanR, gentR	lp5	[45]
Tn::*bb0412*	Tn mutant with Tn insertion in the forward orientation at position 423620 in *bb0412* (insertion ratio 0.73); kanR, gentR	lp5	[45]
Tn::*bb0164*	Tn mutant with Tn insertion in the reverse orientation at position 164997 in *bb0164* (insertion ratio 0.90); kanR, gentR	lp5, cp32-6	[45]
Tn::*bb0050*	Tn mutant with Tn insertion in the reverse orientation at position 47349 in *bb0050* (insertion ratio 0.27); kanR, gentR	cp32-6, cp9	[45]
Tn::*bb0051*	Tn mutant with Tn insertion in the forward orientation at position 48045 in *bb0051* (insertion ratio 0.20); kanR, gentR	lp28-1, lp5	[45]
MR505	Tn::*bb0017* + pMR05 (Tn::*bb0017* expressing *bb0017 -FLAG* from a replicating plasmid); kanR, gentR, strepR	lp5, cp9	This work
MR 506	BS101+ pMH88R (Δ*bb0017* with constitutive expression of *glp* operon)	cp32-6, lp21, lp5	This work
BS101	5A18NP1 + pBS01 (Δ*bb0017)* kanR, strepR	cp32-6, lp21, lp5	This work
BS102	BS101 + pBS102 (SR11’- Δ*bb0017* expressing *bb0017-FLAG* under the control of the native *bb0017* promoter from a plasmid); kanR, gentR, strepR	cp32-6, lp21, lp5, cp9	This work
AK102	Tn::*bb0412* + pAK412 (Tn::*bb0412* expressing *bb0412* at the native *bb0412* locus); kanR, strepR	lp5	This work
JH511	Tn::*bb0164* complemented strain; kanR, gentR, strepR	lp21, lp5	[43]
**Plasmid Name**			
pMR05	pKFSS1 (SacI & XbaI) + PCR fragment containing 298-bp upstream region and *bb0017*FL-FLAG; strepR		This work
pMH88R	pSCB-kan/amp +1.6 kbp fragment upstream of *bb0240* (starting from 205 bp upstream of the *bb0240* ORF + *aacC*		[33]
pBS01	pBlueScript + 3 PCR fragments containing 1835-bp upstream of *bb0017* (chromosomal coordinates 14010-15844), P*flgB*-*aadA*, and 1999-bp downstream of *bb0017*; strepR		This work
pBS02	pMR05, *aadA*::*aacC1*, gentR		This work
pAK412	pBlueScript (XhoI & BamHI) + 3 PCR fragments containing 995-bp upstream, P*flgB*-*aadA*, and 1022 bp downstream of *bb0412*; strepR		This work

* In addition to lp56 and lp28-4

### Feeding of ticks with *B*. *burgdorferi* by immersion

Infection of ticks with *B*. *burgdorferi* from the mutant library was performed using a method previously described [48]. Briefly, before immersion, spirochete cell density was determined by dark field microscopy. Cell suspensions were centrifuged for 10 min at 8,000 x g and were resuspended at the desired cell density and in the desired medium. *I*. *scapularis* larvae were obtained from the National Tick Research and Education Resource at Oklahoma State University and were maintained in a humid tick incubating chamber at room temperature. Larvae were used within 4 months of emergence.

Before immersion, *I*. *scapularis* larvae were removed from the chamber and allowed to sit at ambient humidity in an air-conditioned room for 2h. The larvae were then transferred with a small brush into 1.5 ml microcentrifuge tubes. *B*. *burgdorferi* culture suspension of 108 bacteria in 1 ml was then added to the ticks. For the Tn-seq studies, the suspension consisted of the *B*. *burgdorferi* Tn mutant library [42, 43]. For confirmatory experiments, individual mutants, mixtures of mutants and complemented mutants, or mixtures of mutants and controls were used in the suspension. The tubes were gently vortexed to suspend larvae in the culture and incubated at 32°C for 1 h. Tubes were gently vortexed every 15 min to redistribute ticks in the culture. After incubation at 32°C, tubes were centrifuged at 200 x g for 1 min. The supernatant was removed, and ticks were washed once with phosphate-buffered saline (PBS).

Larvae were then transferred from the microcentrifuge tube to a sealed mouse restraining device. The mice were of C57BL/6 background and were all females aged 4-6 weeks old. A mouse was placed into the restrainer containing the larvae, and the larvae were allowed to attach. Mice were removed from the restrainer after 30 min and transferred to cages suspended over water moats. Engorged larvae were collected from the moats 3 to 5 days after placement and transferred into a tick incubation chamber.

### Tick processing for Tn-seq analysis

Ticks were collected as they completed feeding from the animals. Cages were checked daily to collect ticks. Ticks were batched and processed for Tn-seq up to 48 hrs after collection [48]. Briefly, ticks were washed in 3% hydrogen peroxide, 70% ethanol, and finally in PBS. The ticks were allowed to dry before placement in 500 μl of BSK-II medium with kanamycin and gentamicin. To isolate spirochetes from the ticks, the ticks were crushed with a plastic pestle (Fisherbrand RNase- Free Disposable Pellet Pestles). These tick homogenates were inoculated into 5 ml of BSK-II containing kanamycin and gentamicin. The cultures were incubated for two days to allow outgrowth. Following this, the spirochetes were pelleted by centrifugation for 10 min at 8,000 x g. Bacterial pellets were washed once in PBS, and then the dry pellet was stored at -80°C until further processing for Tn-seq.

### Preparing libraries for Illumina sequencing

Genomic libraries for sequencing were constructed as described previously [42,43]. Chromosomal DNA was sheared using the M220 Focused-ultrasonicator (Covaris) in microTUBEs with a target peak at 350 bp. The first round of PCR amplification was performed using a modified primer with optimized annealing to the Tn (pMargent1A, 5'-ggtaccttaggagaccgggg-3')[43]. Libraries were multiplexed and pooled for analysis. Sequencing was performed on an Illumina HiSeq 2500 at the Tufts University Core Facility as 50-bp single-end reads, as described previously [42,43].

### Tn-seq data analysis

Sequenced reads were clustered by barcode sequence. Data analysis were performed using the Galaxy platform and followed a previously published protocol [43]. We obtained an average of 1.2 x 10^7^ reads per barcode, with 1.7 x 10^6^ reads per condition for analysis after removal of low quality sequences [43]. Reads were mapped to the *B*. *burgdorferi* B31 genome using Bowtie, and a custom script was used to count the number of sequence reads corresponding to each insertion site in the genome. Sequence reads were analyzed “by-site” and “by-gene”. Only Tn mutants that were represented by at least ten sequence reads in both input samples were included in the “by site” analysis. In contrast, the “by-gene” analysis included all sequence reads mapping within a particular gene. Genes represented by less than ten sequence reads in both untreated samples were excluded from the “by-gene” analysis. Tn mutants with zero reads in the output samples were assigned a value of one for the purpose of calculation. The frequency of each Tn mutant in a particular condition was determined by dividing the number of sequence reads corresponding to each Tn mutant by the total number of sequences in the barcode. A frequency ratio was then determined by dividing the frequency of a Tn mutant in the output (bacteria recovered from the ticks) sample by its frequency in the input population. For the purposes of prioritizing mutants for follow-up, frequency ratios between 0.5 and 2 were considered neutral.

### Generation of *B*. *burgdorferi* mutant and complemented strains

A plasmid for complementation of the Tn::*bb0164* mutant was generated previously by overlap PCR [43]. A plasmid for directing *cis* complementation of Tn::*bb0412* via allelic exchange was generated from three PCR fragments: intact *bb0412* with 995 bp upstream sequence (F1), P*flgB*-*aadA* for antibiotic selection (F2), and 1022 bp downstream of *bb0412* (F3). Primers were designed with approximately 30 bp on the 5' end for overlap, with the numerically assigned PCR products assembled in order into the final construct. See S1 Table for a list of all primer sequences used in this study. Individual PCR fragments were amplified with AccuPrime Pfx (ThermoFisher Scientific, MA) per the manufacturer's instructions. An overlap PCR was performed with equal volumes of the appropriate number of PCR fragments with AccuPrime Pfx reagents per the manufacturer's recommendations. Following PCR, the products were resolved by agarose gel electrophoresis and gel-purified (Zymoclean Gel DNA Recovery Kit, Zymo Research, Irvine, CA) for cloning into pCR-Blunt (ThermoFisher Scientific, Grand Island, NY) following the manufacturer's protocol. The *bb0412* complementation vector was designated pAK412 (Table 1).

Plasmid pMR05 was constructed for *trans* complementation of the Tn::*bb0017* mutant was generated by amplifying a DNA sequence containing the *bb0017* open reading frame and the 298-bp upstream region from 5A18NP1 genomic DNA via PCR using the primers bb0017FLAG-F-SacI and bb0017FLAG-R-XbaI (S1 Table). The resulting PCR products as well as pKFSS1 were digested with SacI and XbaI, and the resulting PCR product and pKFSS1 fragments were ligated together using T4 DNA Ligase (New England Biolabs), generating pMR05 (Table 1).

Plasmid pBS01 was constructed to direct allelic exchange at the *bb0017* locus, resulting in deletion of the *bb0017* open reading frame (Table 1). Plasmid pBS01 contains 1835 bp of sequence upstream of *bb0017* (amplified from genomic DNA using primers bb0017del1 and bb0017del2), followed by a sequence containing the constitutive P*flgB* promoter and a streptomycin resistance gene (*aadA*, amplified from pKFSS1 using primers bb0017del3 and bb0017del4), followed by 1999 bp of sequence downstream of *bb0017* (amplified from genomic DNA using primers bb0017del5 and bb0017del6). PCR products were purified using the Qiagen PCR purification kit and were subsequently assembled into the pBlueScript cloning vector using the NEBuilder HiFi DNA Assembly Cloning Kit.

Plasmid pBS02 was constructed to direct expression of *bb0017* in the Δ*bb0017* mutant. Plasmid pBS02 is identical to pMR05, except for replacement of the streptomycin resistance cassette by a gentamicin cassette (Table 1). The streptomycin cassette was excised from pMR05 by restriction digest with AatII and NdeI. The gentamicin cassette had been previously subcloned into the pCR2.1cloning vector. The gentamicin resistance gene was excised from this vector using the same restriction enzymes as above, and the resulting fragment was ligated with the digested pMR05 backbone (Table 1).

Plasmid pMH88R was used to constitutively express the *glp* operon in the Δ*bb0017* mutant. Plasmid pMH88R was a kind gift of Dr. Frank Yang [33]. All completed plasmids were verified by restriction digest and dideoxy sequencing.

Plasmids were introduced into *B*. *burgdorferi* by transformation as previously described [50,51]. *Cis* complementation vector pAK412 was transformed into *B*. *burgdorferi* Tn::*bb0412*, and transformants were designated AK102 (Table 1). The complemented strain was screened by PCR for allelic exchange using the forward primer of fragment 1 and the reverse primer for the P*flgB*- *aadA* cassette for each construct. The *bb0017* deletion construct pBS01 was transformed into *B*. *burgdorferi* strain 5A18NP1, generating strain Δ*bb0017* (Table 1). In order to complement the *bb0017* mutation, plasmid pBS02 was introduced into the Δ*bb0017* background, generating strain Δ*bb0017 + bb0017* (Table 1). The overexpression of the *glp* operon construct pMH88R was transformed into the Δ*bb0017* mutant and was used to generate the Δ*bb0017 + glpFKD* strain (Table 1). Potential transformants were confirmed by PCR with primers designed to detect either a replicating plasmid or a double crossover event, as appropriate, followed by dideoxy sequencing of the PCR product to confirm the expected nucleotide sequence.

### Tick competition assays

To evaluate survival in the tick host, individual Tn mutants of interest were combined with their respective complemented strain or wild type bacteria in a 1:1 mixture. Each strain was grown independently. Cell density was determined by microscopy, and 5 x 10^7^ *B*. *burgdorferi* were harvested by centrifugation. The pellets from both cultures were then resuspended in the same 1 ml of BSK-II medium to a final overall density of 1 x 10^8^ cells/ml. Ticks were submerged in the cultures as described, placed on mice for feeding, and collected over three days as described above. Each tick was washed successively with 3% hydrogen peroxide, 70% ethanol and PBS, then crushed into 250 μl of BSK-II containing kanamycin and 5 μg/ml amphotericin B. The cultures were allowed to acclimate in liquid medium for a period of 2 h before plating, to allow the bacteria to escape from the crushed tick into the medium. Plating in semi-solid agarose overlay was performed as previously described [50]. These plates were then sealed in plastic bags and placed at 32°C for 10 days. The plates were removed from the incubator and colonies were enumerated. The ratio of the wild-type or complemented strains to the Tn mutant was determined by counting the colonies on the appropriate antibiotic selective plates. A competitive index was calculated for these experiments by dividing the amount of mutant recovered by the amount of complement or WT that was recovered. In the case where no mutant was recovered, its value was set to one for the purposes of calculation of the competitive index.

### RNA-sequencing (RNA-seq) library preparation and data analysis

Two independent cultures of the 5A18NP1 and Tn::*bb0017* strains were grown to mid-logarithmic phase, washed once in PBS, and resuspended in BSK II medium at a concentration of 6 × 107 bacteria/ml. Cultures were incubated at 32°C with 1% CO2 for 1 h. The bacterial pellet was harvested by centrifugation at 9500 × *g* for 5 min at 4°C, washed once in ice-cold PBS, and frozen on dry ice. RNA was isolated using the miRNeasy kit (Qiagen). The TURBO DNA-*free* kit (Invitrogen) was used to remove contaminating genomic DNA. Library preparation for RNA-seq analysis was conducted at the Tufts University Genomics Core Facility. Samples were depleted of rRNA using the Gram-Negative Ribo-Zero rRNA Removal Kit (Illumina). Strand-specific libraries were prepared using the TruSeq Stranded mRNA Library Prep Kit (Illumina). Sequencing was performed on an Illumina HiSeq 2500 at the Tufts University Core Facility as 50-bp single-end reads.

Data analysis were performed using the Galaxy platform [43]. Sequence reads were aligned to the *B*. *burgdorferi* B31 genome using TopHat for Illumina v1.4.1 with the default settings. Differences in transcript expression were determined using CuffDiff, again using the default settings.

### Reverse transcriptase quantitative PCR (qRT-PCR)

Total RNA was extracted from bacterial cells grown to mid-logarithmic growth phase at 32°C using TRIzol (Invitrogen) following the manufacturer’s instructions. RNA samples were treated with the TURBO DNA-*free* kit (Invitrogen) to remove contaminating DNA. cDNA was prepared using random hexamers (Promega) and the ImProm-II Reverse Transcription System (Promega). Control reactions were performed in the absence of reverse transcriptase to control for the presence of genomic DNA. Sequences of the primers used to determine the differential expression of target genes are listed in S1 Table, and expression levels were normalized against those of the *B*. *burgdorferi* housekeeping gene *flaB*.

Quantification of target genes from cDNA was performed using the iTaq Universal SYBR Green Supermix (BioRad)[43]. Samples were run in duplicate or triplicate. Analysis of the RT-qPCR data was conducted using the ΔCT method. Data collection was performed using the CFX Connect Real- Time PCR Detection System (BioRad).

### Western blots

Strains were grown in BSK-II at 32°C/1% CO2 until cultures reached early stationary phase. A volume corresponding to 1 × 10^8^ bacteria was harvested by centrifugation at 9500 × *g* for 5 min at 4°C. The bacterial pellet was frozen at -80°C until processing. To lyse the cells, the bacterial pellet was resuspended in approximately 100 μl of 1X NuPAGE buffer (ThermoFisher) and boiled for 5 min. A 20 μl volume of each lysate was electrophoresed in 4-15% gradient SDS-PAGE gels (BioRad). Proteins were transferred to a polyvinyldifluoride (PVDF) membrane (Trans-Blot Turbo BioRad). Membranes were blocked in 5% milk in Tris-buffered saline containing 0.05% Tween-20 (TTBS). Primary antibodies were diluted as follows in TTBS: anti-RpoS (1:50, courtesy of Dr. Frank Yang), anti-BosR (1:500, courtesy of Dr. Frank Yang), anti-FlaB (1:1000, courtesy of Dr. Xin Li), anti-OspC (1:10,000, courtesy of Dr. Xin Li), anti-OspA (1:1000, Rockland Immunochemicals) and anti- DbpA (1:1000, Rockland Immunochemicals). Appropriate horseradish peroxidase (HRP)- conjugated secondary antibodies were used at a 1:10,000 dilution in TTBS. Detection was performed using the Luminata Forte substrate (Millipore), followed by exposure to film (Denville Scientific) or imaging using a ChemiDoc XRS+ Imager.

## Results

### *In vivo* Tn-seq screen in larval ticks

In order to use Tn-seq to determine genes involved in *B*. *burgdorferi* fitness for survival in ticks, we first needed to decide the number of ticks to include in the experiments. Using immersion feeding, it has previously been shown that approximately 103 *B*. *burgdorferi* could be recovered per tick [48,52]. However, these experiments examined a single strain of bacteria, and since bacteria were allowed to replicate within the tick before recovery, the actual number of bacteria entering to establish colonization was likely less than 10^3^. We were also concerned that the bottleneck may be further exacerbated by plasmid loss in the parental strain of the transposon library. It has previously been reported that the mean number of spirochetes per tick with 5A18NP1 lacking both lp28-4 and lp56 plasmids was lower than that in ticks infected with *B*. *burgdorferi* harboring all plasmids [53]. The ordered transposon library contains insertions into 45.5% of the predicted protein encoding genes [45]. To ensure sufficient coverage of the pooled library of around 4,000 mutants we chose to target approximately 150 ticks per experiment. If only half the predicted number of bacteria established infection (500 bacteria per tick), this approach should still provide approximately 20- fold coverage of the mutants within our library.

In each of two independent experiments, approximately 300 larval ticks were immersed in a culture solution containing the entire Tn library and then fed on mice. This resulted in the collection of approximately 160 fed ticks per experiment. Fed ticks were processed and cultured in BSK medium for 2 days. The input culture was also cultured for an additional 2 days to match the tick cultures. Bacteria from both cultures were harvested and sequencing libraries prepared.

**Fig 1:**
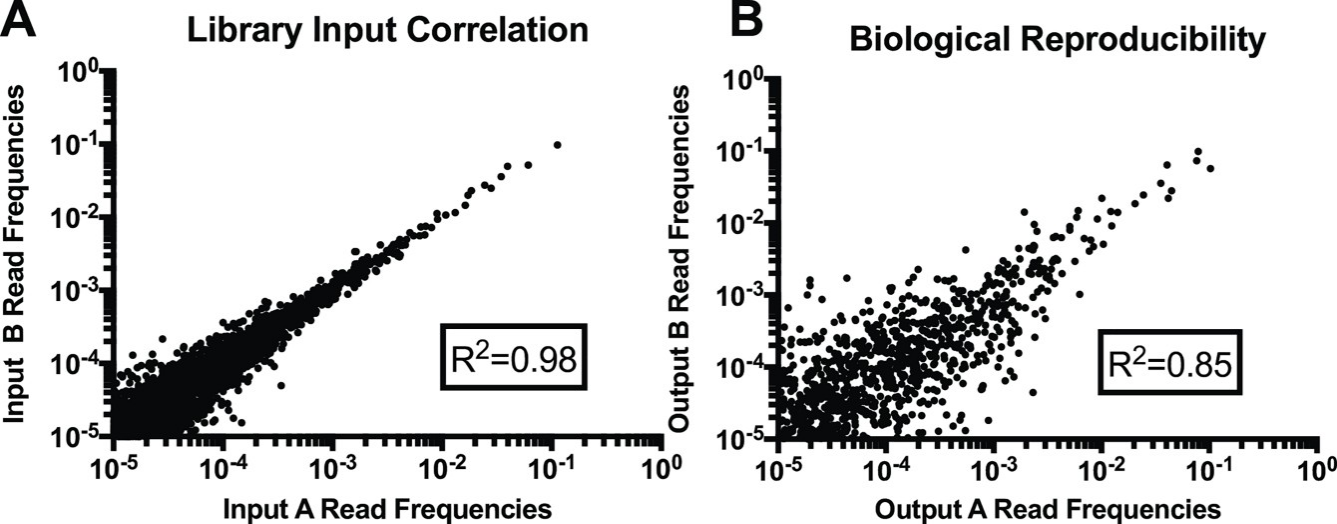
Correlation between replicates of the larval tick survival Tn-seq experiment. (A) Correlation between the mutant compositions of the two replicate cultures of the input Tn library. (B) Correlation between the mutant compositions of the two replicate cultures of the Tn mutants recovered from the fed larvae (output library). Each data point represents the frequency of an individual Tn mutant in relation to the total input or output respectively. Each axis represents a different biological replicate.

Reproducibility was high between the two input libraries (Fig 1A, Pearson coefficient R2=0.98). The correlation between the Tn frequencies of the populations recovered from the two groups of tick larvae was also high with a Pearson coefficient R2= 0.85 (Fig 1B). A frequency ratio was calculated for each Tn mutant in the library by comparing its frequency in the output library to its frequency in the input library. For analysis, we included only Tn mutants represented by at least 10 sequence reads (out of a total of approximately 1.7 x 10^6^ sequence reads per experiment) in both replicates of the input libraries. This number was chosen to reduce the risk of stochastic loss after selection in the ticks. Tn mutants that were represented in the input library but had zero reads in the output library were assigned a value of one read in order to be able to calculate a frequency ratio. A frequency ratio less than one indicates that the Tn mutant decreased in frequency after recovery from fed ticks suggesting that the disrupted gene is involved in survival in the larval host. A frequency ratio greater than one indicates that the Tn mutant increased in frequency after larval colonization suggesting that the disruption of the corresponding gene provides a fitness advantage. An overall frequency ratio was also calculated for each gene by aggregating all of the sequence reads mapping to Tn insertions within the same gene (S1 File). A complete list of mutant fitness for larval colonization by gene and by site is provided in S1 File.

**Fig 2:**
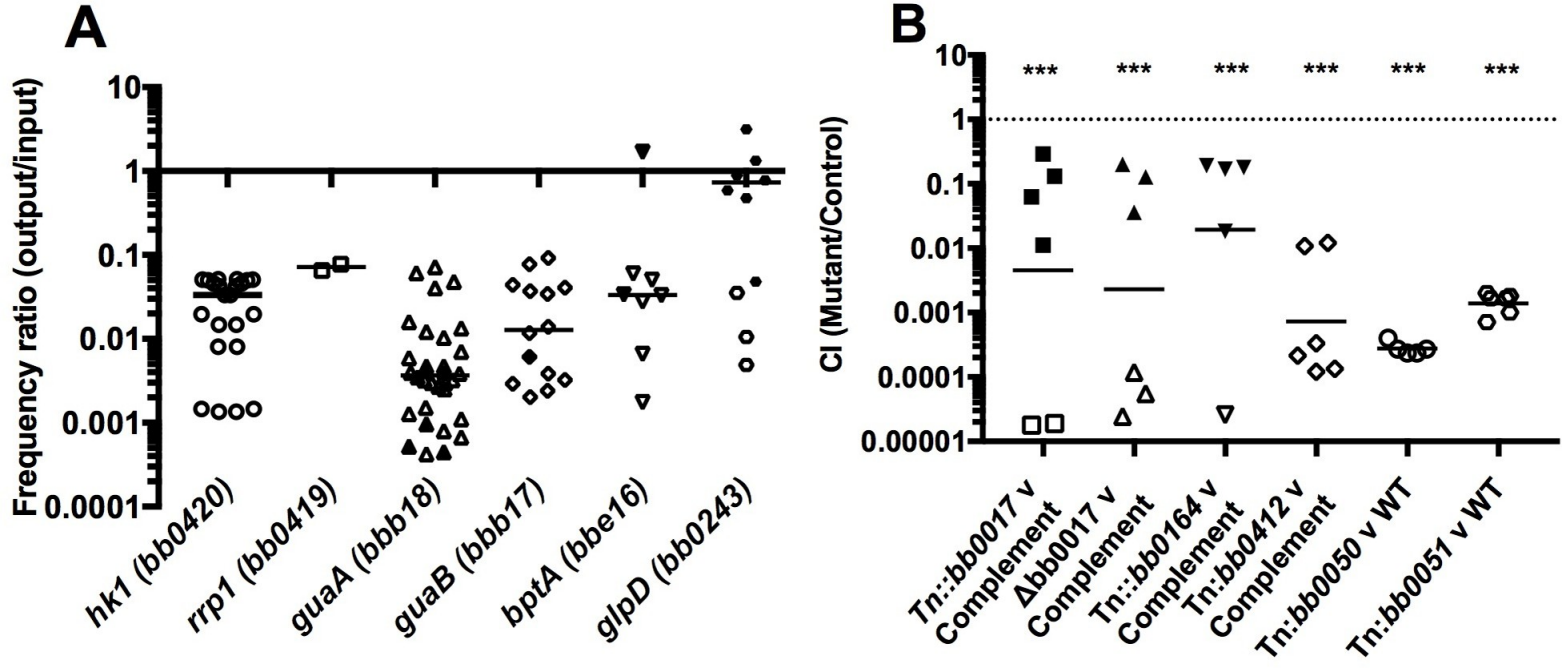
Fitness of selected genes required for *Borrelia burgdorferi* survival in larval ticks. (A) Frequency ratios of individual Tn mutants with insertions into the indicated genes known to be important in *B*. *burgdorfer*i survival in the tick. The frequency ratios of individual Tn mutants with insertions in these genes are shown for both replicates. The median is indicated with a bar. An open shape indicates that no reads were recovered for this mutant and for the purposes of calculation the read number was set to one. (B) Individual Tn mutants were mixed in a one to one ratio with the parental strain 5A18NP1 or the respective complemented strain, and the mixtures were used to infect larvae. After a blood meal these larvae were crushed and plated with appropriate antibiotics to distinguish between the Tn mutant and the complemented strain (or parental strain). Competitive Indices (CI) were determined. Closed shapes indicate that the Tn mutant was recovered following the competition experiment. Open shapes indicate no Tn mutants were recovered following competition experiment. In these cases, the number of Tn mutants recovered was set to one for the purposes of calculation. Statistical significance was determined by one sample t test (***, P<0.0001). (Statistics performed on GraphPad Prism version 7, GraphPad Software, LaJolla California).

In order to validate our screen, we began by analyzing genes that have been previously shown to be essential for tick survival, to ensure that these genes had been identified in the screen. Borrelial genes that have been described in the literature as critical to tick survival for which mutants are present in the library include: *bb0419* and *bb0420*, respectively encoding a response regulator designated Rrp1 and a histidine kinase designated Hk1 [30,35]; *guaA* and *guaB*, two genes involved in the purine salvage pathway [37]; *glpD*, encoding a glycerol 3-phosphate dehydrogenase [33,38]; and *bptA*, a surface-expressed lipoprotein [8]. In the Tn-seq experiment, consistent with previously published results, insertional mutants in each of these genes except *glpD* showed attenuated ability to survive in the tick, with median frequency ratios of <0.1 (Fig 2A). GlpD has been shown to be important following the molt from larvae to nymph when carbon sources are less abundant and the organism begins to utilize glycerol, which may explain why Tn::*glpD* mutants did not exhibit phenotypes in our screen [35].

**Fig 3:**
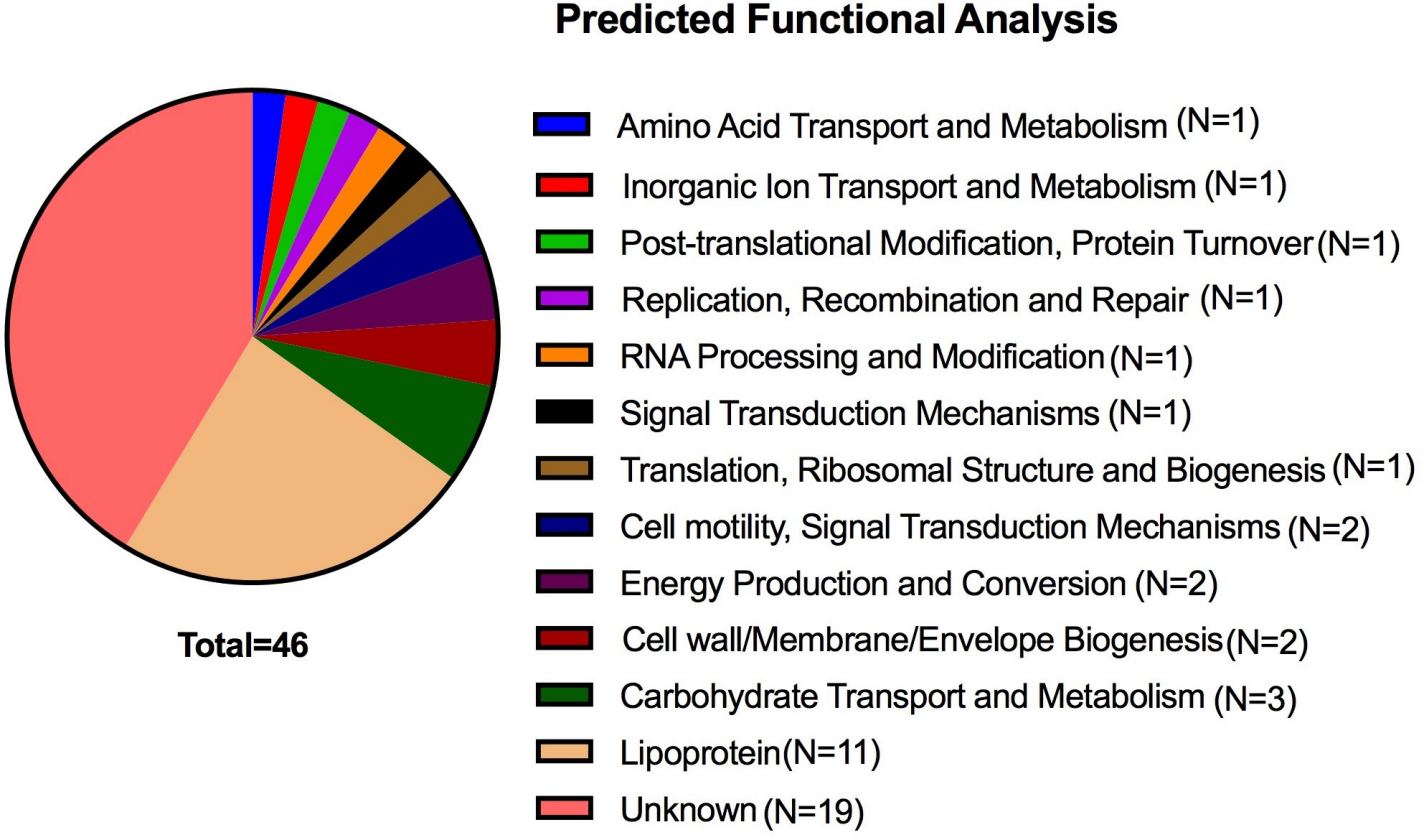
Functional classification of genes required for tick colonization. A cluster of orthologous gene analysis was performed on 46 genes that were found at a frequency of greater than 100 reads in the input libraries and were unrecovered in all output populations. Classifications were based on predicted functions.

Further analysis was performed to identify new genes involved in tick survival. A large number of mutants (N=309) had a frequency ratio of less than 0.5 compared with the input library. In order to prioritize mutants with the strongest phenotypes for follow-up analysis, we focused on mutants with a fitness ratio of less than 0.1. Mutants with insertions in 102 genes had an average overall frequency ratio below 0.1 in both experiments (S1 File). However, this group of 102 mutants included some with insertions into genes that have been shown not to be required for tick colonization (e.g. *ospC*). While many of this group of 102 genes may be involved in tick survival as it includes many of the genes previously identified as involved in tick colonization, to further reduce the chance of false discovery by the screen, we increased the stringency of our criteria and focused on the subset of 46 genes that had >100 reads in the input library but were completely absent in the processed ticks from both experiments (Table 2). These genes would be predicted to have the greatest impact on fitness for survival in larval ticks. Of these 46 genes, many have no predicted function and have not been previously characterized (Fig 3). Approximately 22% of the genes are predicted lipoproteins, while 7% are involved in carbohydrate transport (Fig 3).

**Table 2:** List of *Borrelia burgdorferi* transposon mutants not recovered from larval ticks.

*Locus*	Gene	Product	Classification by COG analysis
*bb0017*		putative lipoprotein	Lipoprotein
*bb0042*	*phoU*	phosphate transport system regulatory protein	Inorganic ion transport and metabolism
*bb0043*		hypothetical protein	Unknown
*bb0051*		hypothetical protein	Unknown
*bb0071*		putative lipoprotein	Lipoprotein
*bb0116*		pts system, maltose and glucose- specific iiabc component	Carbohydrate Transport and metabolism
*bb0138*		hypothetical protein	Unknown
*bb0155*		hypothetical protein	Unknown
*bb0157*		hypothetical protein	Unknown
*bb0159*		hypothetical protein	Unknown
*bb0228*		hypothetical protein	Post-translational modification, protein turnover, and chaperones
*bb0235*	*ychF*	GTP-binding protein YchF	Translation, ribosomal structure and biogenesis
*bb0267*		hypothetical protein	Replication, recombination and repair
*bb0315*		hypothetical protein	Unknown
*bb0322*		hypothetical protein	Unknown
*bb0411*	*nucA*	DNA/RNA non-specific endonuclease	RNA Processing and Modification
*bb0412*		putative lipoprotein	Lipoprotein
*bb0420*	*hk1*	sensory transduction histidine kinase	Signal transduction mechanisms
*bb0524*		inositol monophosphatase	Carbohydrate Transport and metabolism
*bb0525*		hypothetical protein	Unknown
*bb0542*		hypothetical protein	Unknown
*bb0554*		hypothetical protein	Unknown
*bb0555*		hypothetical protein	Energy production and conversion
*bb0597*		methyl-accepting chemotaxis protein	Cell motility, Signal transduction mechanisms
*bb0617*		hypothetical protein	Unknown
*bb0670*	*cheW*	chemotaxis protein CheW	Cell motility, Signal transduction mechanisms
*bb0761*		putative lipoprotein	Lipoprotein
*bb0767*	*murG*	undecaprenyldiphospho- muramoylpentapeptide beta-N- acetylglucosaminyltransferase	Cell wall/membrane/envelope biogenesis
*bb0825*		hypothetical protein	Unknown
*bb0840*		putative lipoprotein	Lipoprotein
*bbA0078*		putative lipoprotein	Lipoprotein
*bbA01*		lipoprotein/ similar to P13	Lipoprotein
*bbA05*		S1 antigen	Lipoprotein
*bbA25*	*dbpB*	decorin-binding protein B	Lipoprotein
*bbA71*		hypothetical protein	Unknown
*bbB01*		acylphosphatase	Energy production and conversion
*bbB05*	*chbA*	chitibiose transporter protein ChbA	Carbohydrate Transport and metabolism
*bbB16*	oppAIV	oligopeptide ABC transporter OppAIV	Amino acid transport and metabolism
*bbH13*		hypothetical protein/ Similar to RepU	Unknown
*bbJ0056*		hypothetical protein	Unknown
*bbJ13*		hypothetical protein	Unknown
*bbK0058*		hypothetical protein	Unknown
*bbM28*	*mlpF*	lipoprotein	Lipoprotein
*bbM39*		hypothetical protein	Unknown
*bbP28*	*mlpA*	lipoprotein	Lipoprotein
*bbU09*		TM2 family protein	Cell wall/membrane/envelope biogenesis

### Confirmation of gene involvement in tick survival

To confirm the results of the Tn-seq screen, we chose mutants with insertions in five genes (*bb0017*, *bb0164*, *bb0412*, *bb0050* and *bb0051*) that showed the strongest fitness defects, and that have not previously been reported to be involved in tick survival. Each of these genes was well represented by insertion mutants in the input library and had an overall frequency ratio of less than 0.1 following the ingestion of a blood meal by larval ticks. Competition assays were conducted to assess the capability of each individual mutant to survive the larval blood meal (Fig 2B).

Three of the transposon mutants were competed against a complemented strain, while the remaining two transposon mutants were competed against the parental strain (Fig 2B). The Tn::*bb0017*, Tn::*bb0164*, Tn::*bb0412*, Tn::*bb0050* and Tn::*bb0051* mutants were all outcompeted by the parental or respective complemented strains, confirming a role for all five genes in blood meal survival (Fig 2B). We were also able to further confirm this phenotype when competing a *bb0017* clean deletion strain against its complemented strain. (Fig 2B). The complemented strain greatly outcompeted the deletion strain confirming the role of *bb0017* in surviving the blood meal (Fig 2B).

### Identifying the role of identified genes in tick survival

The Tn-seq and competition experiments do not distinguish between the possibilities that 1) the identified genes are required for initial entry into the tick during immersion; or 2) they are required for surviving the blood meal taken by the tick. The competition experiment that was described previously was modified so that the ticks were not allowed to take a blood meal after immersion in the culture containing the two competing strains, allowing us to separate fitness defects due to uptake from fitness defects due to blood meal survival. The ticks were crushed after two hours of immersion feeding or kept overnight and crushed 24 hours post-immersion. The relative frequencies of the mutant and complemented or parental strains were then determined as before. However, in contrast to the competitive defect exhibited by all five Tn mutants after the blood meal, we were able to recover all Tn mutants after immersion feeding in equal or greater numbers compared to the WT or complemented mutants. However, in the absence of a blood meal, as expected, the numbers of bacteria were greatly reduced and *B*. *burgdorferi* was not recovered from all individual ticks. The results of these studies are shown in Table 3. These data support a role for these five borrelial genes in surviving changes associated with the blood meal. Also, importantly, because several of these genes have identified roles in ROS resistance and hydrogen peroxide was used in washing the ticks, these experiments confirm that the hydrogen peroxide wash did not affect selection of these mutants.

**Table 3:** Competition in the absence of a blood meal.

Strains in Competition*	Ratio (Tn mutant: Complement or WT) †
Tn::*bb0017 v* Complement	4.75 (19:4)
Tn::*bb0164* v Complement	3.5 (7:2)
Tn::*bb0412* v Complement	1.33 (12:9)
Tn::*bb0050* v WT	1.19 (117:98)
Tn::*bb0051* v WT	1.88 (17:9)

* Strains were mixed in a 1:1 ratio and used to artificially infect larvae. These larvae were processed as described above. However, to asses the ability of the strains to enter the tick, the larvae were not given a blood meal. Instead they crushed and plated on selective antibiotics at 2 h or 24 h post-infection

† The ratio represents the aggregated results from 18 individual ticks for each competition. These results include the plating experiments that were done at 2 h and 24 h post-infection. The parenthetical values are the actual number of colonies counted.

### *In silico* analysis predicts BB0017 is a membrane-associated signal transduction protein

To begin to better understand the mechanisms by which the genes identified in the Tn-seq screen contribute to tick-phase survival, we performed further investigation into one of the genes identified as critical for survival of the blood meal: *bb0017*. The gene *bb0017* was previously identified in a screen for genes that confer resistance to ROS [43]. BB0017 is highly conserved among both *B*. *burgdorferi* sensu stricto and other sensu lato strains (>99% and >94% identity at the amino acid level, respectively). BB0017 homologues are also conserved in the relapsing fever strains (>80% identity) [54]. In the *B*. *burgdorferi* strain B31, *bb0017* is annotated an integral membrane protein of the YitT family. BB0017 contains four predicted transmembrane domains as well as a C-terminal soluble domain and contains the conserved domain of unknown function DUF2179 (S1A & S1B Fig) [55]. A structure-based similarity search using Phyre2 suggested that the C-terminal domain of BB0017 is structurally similar to PII and PII-like proteins, despite low overall sequence identity (<27% identity) [56]. No high confidence predictions were made for the N-terminal domain of BB0017.

PII proteins are a broadly conserved class of signal transduction proteins found in bacteria, archaea, and plants and are generally small cytoplasmic proteins involved in nitrogen metabolism [57–59]. PII proteins generally function as trimers and control the activity of their regulatory targets through direct protein-protein interactions in response to both post-translational modifications (such as uridylylation) and ligand binding (including ADP, ATP, and 2-oxoglutarate). The long, flexible T- loop mediates interactions with regulatory targets, while a conserved motif in the shorter B-loop is involved in ligand binding (S1C Fig, GlnKEc). More recently, several PII-like families of proteins have been identified in bacteria, including a family of proteins in Gram-positive bacteria that bind cyclic diadenylate monophosphate (c-di-AMP) as well as a broadly conserved family of proteins (CutA) that confer copper tolerance in *Escherichia coli* and bind acetylcholinesterase in mammals [60–66].

While the PII and PII-like proteins share a common ferredoxin-like fold, the lengths of the T and B loops differ significantly between the different protein families. In the case of the PII-like c-di-AMP binding proteins, the lengths of the B and T loops are reversed relative to the PII proteins and are referred to as the B´ and T´ loops (PstASa, S1C Fig). Structural data suggests that the functions of the B´ and T´ loops are also reversed relative to the PII proteins, with the short T´ loop being involved in ligand binding and the long flexible B´ loop possibly involved in effector binding [61,62,64]. In the case of the copper tolerance protein CutA1, both the B and T loops are truncated, and the same is true for BB0017. Interestingly, BB0017 appears to lack conserved residues involved in ligand binding by both the PII and PII-like protein families. The presence of the N-terminal transmembrane domain also distinguishes BB0017 from the PII and PII-like protein families and suggests that membrane localization may be important for BB0017 function.

### BB0017 downregulates expression of mammalian-phase lipoproteins

**Fig 4:**
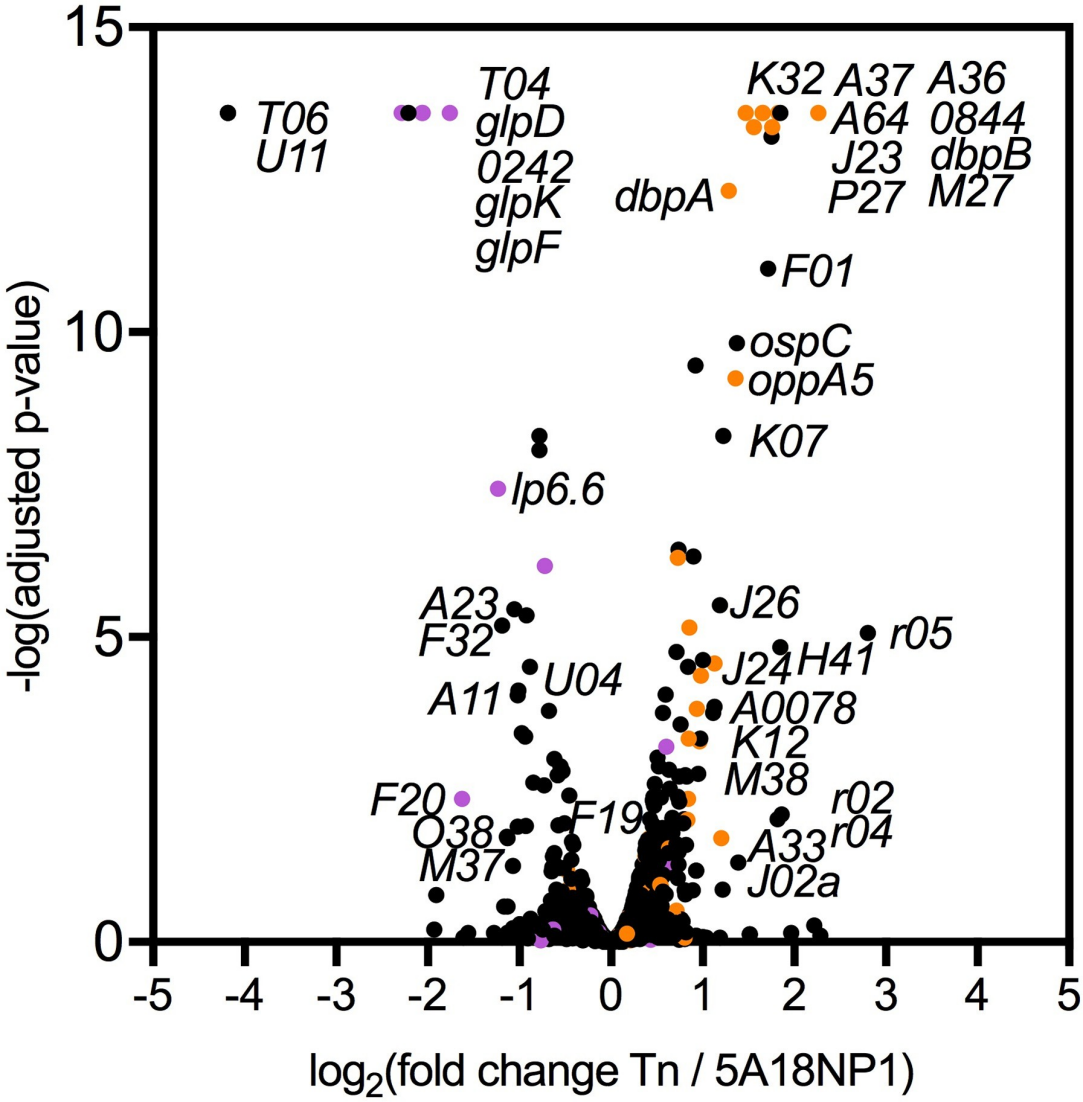
Genes differentially expressed in the Tn::*bb0017* mutant compared to parental strain in RNA-seq experiment. Volcano plot showing differential gene expression between the Tn::*bb0017* mutant and the parental strain. Genes that were significantly different are labeled by name in the graph. Purple circles indicates that the gene is repressed by *rpoS* while orange circles indicates that a gene is activated by *rpoS*. The gene abbreviation system assigns a letter based on the plasmid location.

Because BB0017 contains a putative signal transduction domain, we hypothesized that the Tn::*bb0017* mutant would exhibit global differences in gene expression compared to the parental strain. Total RNA was isolated from the parental and Tn::*bb0017* strains, and RNA sequencing (RNA-seq) was used to compare the transcriptomes of both strains. We identified 16 genes that were significantly downregulated more than twofold in the Tn::*bb0017* mutant compared to the parental strain and 25 genes that were significantly upregulated more than twofold (S2 Table and Fig 4). It is important to note that *bb0017* does not appear in S2 Table. While expression of *bb0017* was significantly different between the Tn::*bb0017* and parental strains, the difference was less than twofold, and *bb0017* expression was actually higher in the Tn::*bb0017* mutant compared to the parental strain. Sequence coverage maps confirm that transcription in the Tn::*bb0017* mutant is abrogated downstream of the Tn insertion as expected (S2 Fig). However, increased numbers of sequence reads mapped in the 5’ portion of *bb0017*, likely due to transcription from the strong P*flaB* promoter contained within the Tn (S2 Fig). It is unclear whether there is translation of the 5’ portion of *bb0017* in the Tn::*bb0017* mutant, resulting in a truncated protein, but if a truncated protein is produced in the Tn::*bb0017* mutant, these results could suggest that the C-terminal portion of BB0017 is the critical portion for survival in the tick.

Strikingly, the putative *bb0017* regulon overlaps significantly with that of RpoS, a key regulator of virulence gene expression in *B*. *burgdorferi* [15,16,19,20]. RpoS is directly responsible for upregulating a number of genes required for survival in the mammalian host, including *dbpA*, *dbpB*, *ospC*, and *bbk32* and repressing expression of genes important for tick survival such as *glpD* [15,19,20,38,67]. The *dbpA*, *dbpB*, *ospC*, and *bbk32* genes are all upregulated in the Tn::*bb0017* mutant (S2 Table, Fig 4). Several genes known to be subject to RpoS-mediated repression, including genes located within a glycerol utilization operon important for tick infectivity, are downregulated in the Tn::*bb0017* mutant (*bb0240*-*bb0243*, S2 Table) [38]; however, other regulators such as c-di-GMP may also affect expression of these genes.

### RNA-seq validation

To confirm the results of the RNA-seq screen, we generated a mutant lacking the entire *bb0017* open reading frame. A survey of available *B*. *burgdorferi* genome sequences revealed two different annotated start sites (S1 Fig). We chose to delete the region encompassing the first start site, which includes a putative 71 bp small RNA (SR0011) in the *bb0016-bb0017* intergenic region [68] (S1 Fig). We restored *bb0017* expression including the upstream SR11 intergenic region under the control of the native promoter from a replicating plasmid in the Δ*bb0017* mutant, and confirmed expression by qRT-PCR (S3 Fig).

**Fig 5:**
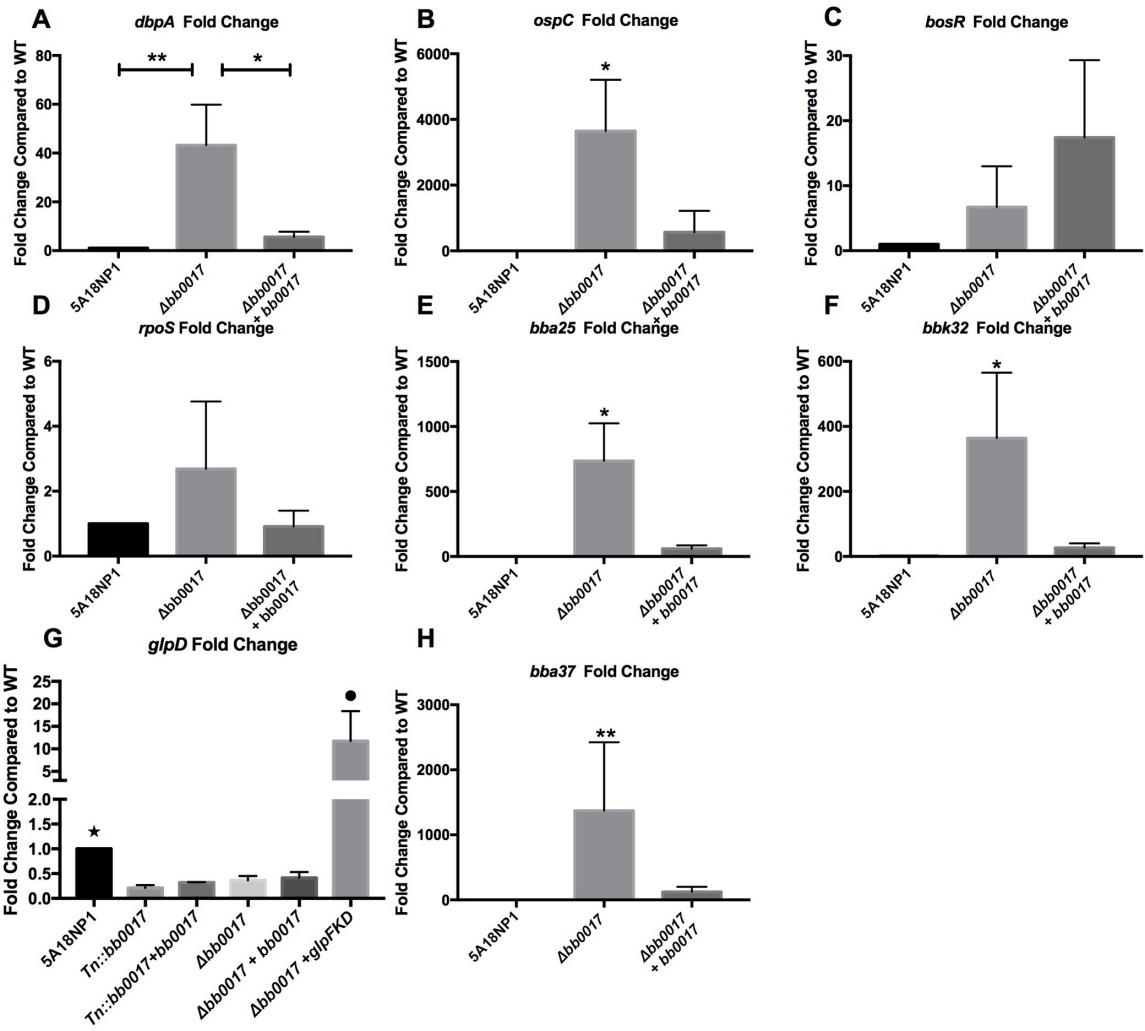
Validation of RNA-seq by RT-qPCR. RT-qPCR for expression of a panel of genes that were of significance from the RNA-seq in the parental strain 5A18NP1, the Δ*bb0017* mutant, and the respective complemented strain. A) *dbpA*, B) *ospC*, C) *bosR*, D) *rpoS*, E) *bba25*, F) *bbk32*, G) *glpD*, H) *bba37*. Expression of the target genes was normalized to expression of the *B*. *burgdorferi* housekeeping gene *flaB* using the ΔCT method. Statistics were performed using one way ANOVA with Tukey’s test for multiple comparisons.(*= P<0.05, **= P<0.005)(Black Star= P<0.05 from parental, Black circle= P<0.05 from *glpFKD* over expressing strain) (Statistics performed on GraphPad Prism version 7, GraphPad Software, LaJolla California).

We performed qRT-PCR on the Δ*bb0017* mutant as well as the complemented strain to validate the results of the RNA-seq using the transposon insertion strain. We selected a subset of differentially regulated genes from the RNA-seq, as well as some representative regulatory proteins of *B*. *burgdorferi* that showed no change in expression. *bosR* and *rpoS* levels were not significantly different in the RNA-seq and this phenotype was reproduced in the deletion strain as well as the complement by qRT-PCR (Fig 5). We then confirmed six genes, *ospC*, *dbpA*, *glpD*, *bba37*, *bba25*, and *bbk32* that were differentially expressed by RNA-seq in the transposon mutant strain. Each showed a similar pattern of expression in the clean deletion strain with recovery in the complemented mutant strain, with the exception of *glpD* (Fig 5). Transcription of *glpD* was decreased in the transposon mutant and its complement as well as the deletion strain and its complement in comparison to the wild type making it unlikely that this difference was due to secondary site mutations or polar effects as each of the mutants and complements were created from separate isolations from the parental strain. Of note is that all the changes are small (fourfold) compared to the other genes tested by qRT-PCR. Given the lack of involvement of *glpD* involvement in tick survival as assayed by the Tn-seq and its established role at a different stage in tick survival, it is likely that the change is not physiologically relevant.

### Overexpression of the glycerol utilization operon in the Δ*bb0017* mutant does not restore the ability to survive the blood meal

To ensure that we were not missing a role for GlpD in mediating effects of the Δ*bb0017* mutant, we created a strain that overexpresses the entire *glp* operon, including *glpFKD*, in the Δ*bb017* deletion strain. This construct has previously been used to successfully overexpress GlpD [33]. We confirmed that *glpD* was successfully transcriptionally over-expressed by qRT-PCR (Fig 5). Using this strain, we then performed a competition experiment between the *glp operon* overexpressing strain and the Δ*bb0017* strain. We were not able to recover either strain from this experiment following selective plating from six collected fed larvae. This indicates that increasing the ability for glycerol utilization is not sufficient to rescue the Δ*bb0017* mutant and the defect in tick colonization is unlikely to result strictly from decreased expression of *glpD*.

### BB0017 represses lipoprotein expression in a BosR-and RpoS-dependent manner

**Fig 6:**
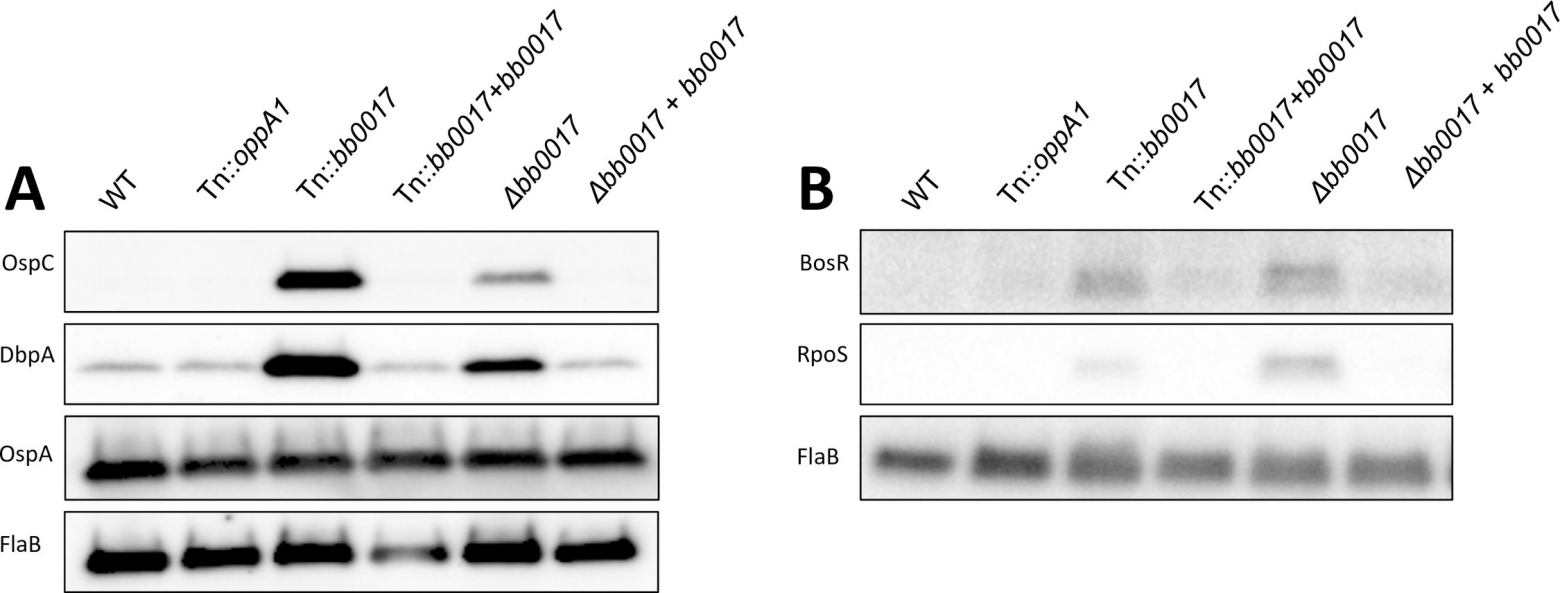
**Immunoblot analysis of** (A) lipoprotein expression and (B) regulatory protein expression across different *B*. *burgdorferi bb0017* mutant and complemented strains. A Tn::*oppA1* mutant was also included in these experiments as a further control strain. Bacterial cultures were grown at 32°C with 1% CO2 until early stationary phase. A volume corresponding to 1×10^8^ bacteria was harvested by centrifugation, cells were washed and lysed, and lysate corresponding to approximately 2×10^5^ cells was subjected to immunoblot analysis. FlaB levels were analyzed as a loading control.

We performed immunoblots for OspC and DbpA in the Tn::*bb0017* and Δ*bb0017* mutants. Levels of both DbpA and OspC were elevated in the Tn::*bb0017* and Δ*bb0017* mutants, confirming the results of the RNA-seq screen (Fig 6A). Restoration of *bb0017* expression from a replicating plasmid (which also contains SR0011) in both mutants decreased DbpA and OspC levels to those of the parental strain (Fig 6A). The fact that both the Tn::*bb0017* mutant (in which SR0011 remains intact) and the Δ*bb0017* mutant (in which SR0011 is disrupted) exhibit increased lipoprotein expression, suggests that *bb0017* is required for the phenotype, although these results to do not exclude the possibility that SR0011 may also be involved. Expression of OspA, a surface lipoprotein required for infectivity in the tick, was not affected by the absence of *bb0017* under the conditions tested.

The expression of *dbpA* and *ospC* requires the alternative sigma factor RpoS [15,20]. The regulation of *rpoS* in turn involves a second alternative sigma factor RpoN (σ54), the enhancer binding protein Rrp2, the *Borrelia* oxidative stress response regulator BosR, and the small regulatory RNA DsrA [15,16,24,25,69,70]. We hypothesized that BB0017 mediates the repression of *ospC* and *dbpA* indirectly by affecting the expression of an upstream regulator. We therefore investigated RpoS levels in the Tn::*bb0017* and Δ*bb0017* mutants. RpoS levels were increased in both mutants, and restoration of *bb0017* expression resulted in decreased RpoS levels (Fig 6B). To understand the mechanism by which BB0017 affects RpoS expression, we next investigated production of BosR, a positive regulator of RpoS [24,70]. As was the case for RpoS, BosR levels were increased in the Tn::*bb0017* and Δ*bb0017* mutants, and complementation of *bb0017* restored levels to those similar to the parental strain (Fig 6B).

## Discussion

In this paper, we report the use of massively parallel, next generation sequencing technology to identify genes important in survival in the larval tick host. This study represents the most complete survey of *B*. *burgdorferi* genes that are required for tick survival performed to date and greatly increases our understanding of this critical phase of the *B*. *burgdorferi* life cycle. We have identified many genes that have not previously been associated with tick survival, confirmed the involvement of a subset in tick survival, and began to characterize a mechanism of action for one of the genes, *bb0017*. Notably, because we were able to quickly and inexpensively screen large numbers of ticks, we were able to minimize bottleneck issues that have arisen in other animal studies, and our results showed a high level of experimental reproducibility. The robustness of the technique is exemplified by our ability to identify genes known to be required for tick-phase survival and our ability to validate the phenotypes of all five mutants we selected for further analysis.

There are several caveats in the interpretation of the Tn-seq screen data. First, the transposon library is not saturated and does not contain insertions into all non-essential genes. Mutants for several genes known to be important in tick colonization are not included in the library. Next, the transposon library we used for this study was generated in the 5A18NP1 background, which is missing two plasmids, lp56 and lp28-4. It is possible that the loss of the genes on these plasmids affects the requirement for certain genes or that the regulatory patterns are altered in their absence. Finally, the mechanism we used to infect the tick larvae, immersion feeding, is artificial and may lead to identification of genes that are not involved in natural transmission or, more likely, miss genes that are involved.

A more general caution about screening techniques such as Tn-seq is that the false discovery rate is dependent upon the stringency of the analysis used. We analyzed the data in two tiers. Using our most stringent criteria of no mutants isolated in either replicate, we did not detect any mis-identification in the subset of genes that were confirmed by additional experimental testing. Using slightly less stringent criteria of a 90% decrease in fitness, we already noted some false identifications of genes that have been previously shown to not be involved in tick survival including *ospC*. As with any screen, the goal is to enrich the identification of genes that are actually involved in a process while minimizing false identifications, but regardless of the stringency of the criteria, each of the genes will still need to be confirmed by additional testing. There was quite a bit of variability in frequency between genes that were not completely absent. This occurs because of stochastic loss of mutants due to bottleneck issues that can result in differences in the recovered mutants. One way to minimize this variability is to perform more experiments, which in our case, would mean adding more ticks for each replicate. By averaging results over more experiments, stochastic variability will decrease and we would have increased ability to identify genes with partial fitness impacts. At the numbers of ticks we used, the greatest confidence is for an extreme phenotype.

Understanding these caveats, we identified 46 genes whose disruption resulted in complete loss of Tn mutants from the population following colonization of larval ticks. Of these 46 genes, almost all have not previously been reported to be involved in survival in the tick, and thus the current study represents a significant advancement in our understanding of the genetic factors required for *B*. *burgdorferi* survival in the tick. Of note, many of the tick-phase genes we identified encode membrane-localized lipoproteins, and a significant portion (14 genes, including the five we selected for follow-up analysis), have been previously identified as important for resistance to reactive oxygen and nitrogen species (Table 2)[43].

We confirmed the phenotype for five of the novel tick-phase genes (*bb0017*, *bb0164*, *bb0412*, *bb0050*, and *bb0051*) by individual competition assays for survival in the tick following a blood meal (Fig 2B). Prior to our study, relatively little was known regarding the functions of these gene products, other than their predicted role in ROS resistance. All five gene products are predicted to be membrane-localized, and BB0164 has previously been shown to be involved in controlling intracellular manganese homeostasis [43]. To better understand whether these genes aid 1) the entry of *B*. *burgdorferi* into tick larvae during artificial infection, or 2) bacterial adaptation as the tick takes a blood meal, additional competition studies were carried out following the culture immersion step, but before the blood meal. All of the mutants tested survived as well as (or better than) the controls in the competition assays, suggesting that these genes are involved with survival of the blood meal and not with entry into the tick. That survival of the blood meal poses the larger barrier is not surprising. The blood infusion that the *B*. *burgdorferi* encounters in the midgut of the tick during feeding creates a rapidly changing environment for the spirochete. During the blood meal, there are changes in pH and temperature and exposure to reactive oxygen species (ROS), natural antibodies, and components of complement that can mediate spirochete killing [71–78]. Our results suggest that the ability to resist oxidative stress is likely critically important for survival in the tick host.

We performed further investigations to better understand the mechanisms by which one of the genes identified in our screen, *bb0017*, contributes to survival in the tick. Our *in silico* analysis suggested that BB0017 is part of a larger family of PII and PII-like proteins. However, there are some notable differences that distinguish BB0017 from these protein families, including differences in the lengths of two key loop regions (B/B’ and T/T’) and absence of key conserved residues involved in ligand binding. Thus, if BB0017 does bind ligands such as copper or c-di-AMP as has been previously shown for PII and PII-like proteins, it does so via a unique mechanism. c-di-AMP is produced in *B*. *burgdorferi*, although its potential function as a second messenger in this organism remains unclear [79,80]. It is certainly possible that the true ligand for BB0017 is a different molecule as there are significant differences between BB0017 structure and the structure of other P_II_ proteins. Given the downstream effects of BB0017, it is tempting to speculate that it may bind c-di-GMP, which plays a critical role in *B*. *burgdorferi* gene regulation, however identification of binding of other molecules by BB0017 requires further experimentation.

RNA-seq analysis revealed that interruption of *bb0017* by the Tn insertion results in significantly higher levels of transcription of the genes encoding DbpA and OspC; these results were corroborated by qRT-PCR as well as by immunoblot analyses using strains that had a complete deletion of *bb0017* (S2 Table, Fig 5 and Fig 6A). This regulatory effect in the mutant appears to be mediated by increased levels of BosR and RpoS suggesting that BB0017 acts as a potential negative regulator of these important pathways. Repression of genes highly expressed during mammalian infection would be consistent with a role for BB0017 in tick colonization. Previous studies have shown that expression of *B*. *burgdorferi* genes that are required for one host may result in a fitness defect in colonization of the other host [69,73,76,77,81–83]. The effects of deletion of *bb0017* on RpoS are also likely to affect expression of genes in the *glp* operon as seen by the RNA-seq studies (S2 Table). However, altered expression of the *glp* operon does not appear to account for the survival defect of the *bb0017* mutant in our Tn-seq experiments as overexpression of the *glp* operon was not sufficient to restore the ability to survive the blood meal in a Δ*bb0017* background. This is consistent with the fact that we did not observe a fitness defect for the Tn::*glpD* mutant and that prior studies have shown that the *glp* genes are required at a time point later in the tick cycle than was evaluated in our study [33,38].

The elevated lipoprotein expression profile observed in the *bb0017* mutant is strikingly similar to the phenotype of a mutant lacking the BmtA manganese (Mn) transporter. The *bmtA* mutant exhibits decreased intracellular Mn concentrations, which was shown to result in increased levels of *ospC* expression [84]. In the case of the *bmtA* mutant, the increased *ospC* expression is due to an increase in BosR protein levels at the post-transcriptional level, leading to increased transcriptional activation of RpoS [84]. The post-transcriptional regulation of BosR has also been observed in conditions where CO_2_ is limiting [85]. Our RNA-seq analysis suggests that *bosR* and *rpoS* transcript levels are similar in the Tn::*bb0017* mutant and parental strains, despite the increase we observe in protein levels (Figs 5 and 6) suggesting that BB0017 affects BosR at the post-transcriptional level. In the *bb0017* mutant, RpoS also appears to be upregulated at the post-transcriptional level, suggesting that some mechanism other than direct transcriptional activation by BosR is responsible for increased RpoS levels. There is also precedent for post-transcriptional regulation of RpoS, both in *B*. *burgdorferi* and in other bacteria [69].

The reciprocal expression of two sets of genes required for survival in the tick or mammalian hosts in response to a variety of environmental signals is paradigmatic to borrelial pathogenesis. However, the mechanisms by which these external stimuli are sensed remain to be fully characterized [69,73,76,77,81–83]. Given that BB0017 is predicted to be a membrane-localized signal transduction protein, we hypothesize that this protein may sense changes in the environment to regulate downstream effectors accordingly. The nature of the external signal, if any, sensed by BB0017 remains unclear, although it is likely not Mn. We previously showed that Mn levels are similar in the Tn::*bb0017* mutant compared to the parental strain [43]. Given that PII and PII-like proteins generally affect downstream targets at the post-transcriptional level via direct protein- protein interactions, we predict that the BB0017 regulon may be larger than the list of genes identified in the RNA-seq analysis.

In conclusion, we have found that Tn-seq is a powerful tool in identifying *B*. *burgdorferi* genes important for fitness in surviving the blood meal. We have identified a large number of previously uncharacterized genes involved in the survival of the bacterium in its tick host. These results may provide important new avenues for exploration and understanding how the bacterium adapts to its different hosts. To this end we have further investigated the role of one of the genes identified, *bb0017*. We propose that BB0017 is a potential global regulator in *B*. *burgdorferi* that affects resistance to oxidative stress, survival in the arthropod host, and expression of key virulence determinants. As several of the other genes identified as important for survival of the bacteria during the early stages of tick larval infection also have been identified in prior screens for genes of ROS resistance, our results suggest the importance of ROS resistance in the initial colonization and persistence during the acquisition of the blood meal. Future Tn-seq screens can be tailored to identify genes required for survival during other parts of the bacterial lifecycle within the tick host. This approach will allow investigators to map the network of adaptations used by the bacteria to complete its life cycle.

## Supporting information

S1 Table Sequences of oligonucleotides used in this study. (DOCX)

S2 Table Genes differentially expressed at least 2-fold in the Tn::*bb0017* mutant compared to the parental strain. Rep., replicon; RPKM, Reads Per Kilobase Million; q, False Discovery Rate adjusted p-value; P, parental strain * Repressed by RpoS 20.† Activated by RpoS 19,20,‡ Shading indicates genes with identical or near-identical sequence.
(DOCX)

S1 Fig *In silico* analysis of the *bb0017* locus. (A) Genomic context of *bb0017*. Chromosomal coordinates in the *B*. *burgdorferi* B31 genome are indicated below the genes. The putative sRNA SR0011 identified in the *bb0016-bb0017* intergenic region is shown [69]. Black circles indicate the two annotated *bb0017* start sites at positions 15845 and 15995. Triangles indicate the approximate locations of all Tn insertions present in the *B*. *burgdorferi* Tn library [43,45]. Triangles located above the gene represent Tn insertions on the positive strand, while triangles located below the gene represent Tn insertions on the reverse strand. Shading indicates the median frequency ratio for a particular Tn mutant following exposure to H2O2 [43]: dark red: frequency ratio < 0.25; light red: frequency ratio <0.5; yellow: frequency ratio <1. (B) Predicted membrane topology of BB0017 [55]. TM, transmembrane domain; SOL, soluble domain. A conserved domain of unknown function (DUF2179) is shown. (C) Amino acid alignment of the soluble domain of BB0017 (BB0017SOL) with the PII-like protein PstA from *Staphylococcus aureus* (PstASa), the PII protein GlnK from *Escherichia coli* (GlnKEc), and the PII-like CutA protein from *E*. *coli* (CutA1Ec). The predicted secondary structure of BB0017SOL is indicated [56], along with the known secondary structures for PstASa (PDB code 4D3G), GlnKEc (PDB code 1GNK), and CutA1Ec (PDB code 1NAQ) [65]. Beta sheets (blue shading) and alpha helices (green shading) are indicated. Conserved residues important for ligand binding or regulatory function in the different protein families are shown in red type. For GlnKEc, these residues include the highly conserved Gln-39 and Lys-58 residues involved in salt bridge formation, the Tyr-51 site of uridylylation, and the TGxxGDGKI motif involved in ATP binding [58]. For PstASa, these residues include the highly conserved Thr-28, GGFL motif, and Asn- 31 residue involved in c-di-AMP binding [64]. For CutA1Ec, these residues include Cys-16, His-83, and His-84 involved in copper binding [63].
(TIF)

S2 Fig RNA-seq coverage track showing the sequencing read depth at the *bb0017* locus for two biological replicates of the parental and Tn::*bb0017* strains. The location of the transposon insertion on the reverse strand is indicated with a triangle. The red/green lines in the Tn::bb0017 mutant represent SNPs in the RNA-seq reads relative to the genome sequence. (A= green, C =blue, G yellow, T red).
(TIF)

S3 Fig *bb0017* expression in mutant and complemented strains. qRT-PCR for *bb0017* expression in the parental strain 5A18NP1 (P), the Tn::*bb0017* mutant, a *bb0017* deletion mutant (*bb0017*), and strains in which expression of or *bb0017* was restored in the *bb0017* mutant backgrounds. Expression of *bb0017* was normalized to expression of the *B*. *burgdorferi* housekeeping gene *flaB* using the ΔCT method.
(TIF)

S1 File Results of larval tick Tn-seq experiment. Overall frequency ratios for all genes in *Borrelia burgdorferi* after a blood meal (Excel Tab 1). Frequency ratios of individual Tn mutants after a blood meal (Excel Tab 2). Tn mutants were removed from the analysis if they were not represented by at least 10 sequence reads in both untreated libraries. Zeroes in the treated samples were changed to 1 before calculating frequency (freq) ratios.
(XLSX)

S2 File Genebank file. This is the genebank file used for TN-seq protocols. The file contains all accession numbers to the sequences used for generation of the input file for bowtie.
(GBK)

S3 File Fasta file. This is the fasta file used for TN-seq protocols. This file contains the entire DNA sequence used to assemble the genome for TN-seq analysis pipeline.
(FA) Click here for additional data file.
