# Supplementary material for: Genome-wide screen identifies novel genes required for Borrelia burgdorferi survival in its Ixodes tick vector
Source: PLoS Pathog. 2019 May 14;15(5):e1007644. doi: 10.1371/journal.ppat.1007644 (PMC6516651; doi:10.1371/journal.ppat.1007644)
Supplement: S1 Table — (DOCX) [file ppat.1007644.s001.docx]

| **Oligo name** | **Oligo sequence (restriction sites underlined)** |
| --- | --- |
| bb0017FLAG-F-SacI | GGTGGTGAGCTCGCAAATCTTGCATAATTCATCG |
| bb0017FLAG-R-XbaI | GGTGGTTCTAGATTACTTGTCGTCATCGTCTTTGTAGTCACCGCCACCAATTTCTT GTTTTAATGAG |
| bb0017del1 | CCACCGCGGTGGCGGCCGCTCTAGAACTAGTCTTATGGAATTCAGCTTGTTG |
| bb0017del2 | GGAAATCTTCCTTGAAGCTCGGGTACTTAATGTTAGAGCCACTTTTAAGC |
| bb0017del3 | GCTTAAAAGTGGCTCTAACATTAAGTACCCGAGCTTCAAGGAAGATTTCC |
| bb0017del4 | CTTTTTTTAAGAGATTTTTAATTTTATTTGCCGACTACCTTGGTGATCTCGCC |
| bb0017del5 | GGCGAGATCACCAAGGTAGTCGGCAAATAAAATTAAAAATCTCTTAAAAAAAG |
| bb0017del6 | CCTGCAGCCCGGGGGATCCACTAGTTCCTACTGAAAAATTGTTTATTGAG |
| bb0017-qPCR-F4 | GCCGCTACATTAATAGACGGAA |
| bb0017-qPCR-R4 | CGGTAATGAAACAATTTGGATCAAC |
| bb0243-qPCR-F | CCTTCCCAAGGTAGCCATTTAG |
| bb0243-qPCR-R | GTGCTTCCACAAACAACACTATC |
| bb0409_0412xhoI F | ATCTCGAGGGCAATTCCTTTAATGGGG |
| overlap bb0412_strep R | *CAGGGAAAATTTTCTTTTACTGGCTATAATACCCGAGCTTC* |
| overlap bb0412_strep F | TACTGGCTATAATACCCGAGCTTCAAGGAAGATTTC |
| overlap strep_bb0413 R | AACTTCTTTTTTTTATTTGCCGACTACCTTGGTG |
| overlap strep_bb0413 F | GTCGGCAAATAAAAAAAAGAAGTTAAAAAATAGGACGAAGCT |
| bb0414 bamHI R | ATGGATCCATTTGTGATGGGTTTTCAAGAG |
| 0409_0412 xhoI F new | ATCTCGAGGGCAATTCCTTTAATGG |
| Overlap 0412_strep R new | GAAGCTCGGGTATTATAGCCAGTAAAAGAAAATT |
| 0408 F | GGGGGTTTTCTTCAGACCACT |
| 0414 R | TTGGAAGCTTTGATTTGAGTACAA |
| Rpos QPCR F | TCAACCTATCTCCTGCTCAGT |
| Rpos QPCR R | TCA GAT CCC TCT ATT TCT TTG TCC A |
| BB_0771 QPCR F | TGG CAA GCA AAT AAA TCA AT |
| BB_0771 QPCR R | TGC TCC AAT GAA TTT TAC CT |
| GlpD QPCR F | TCC CAA GGT AGC CAT TTA GTA GTC A |
| GlpD QPCR R | TGC TTC CAC AAA CAA CAC TAT CA |
| BB_A37 QPCR F | TGA TTC TCA CAC AGT AAG TG |
| BB_A37 QPCR R | ATC TCT TCT TCG GCT GAT TT |
| BB_K32 QPCR F | AGT CAA TAA CGC AAA GAG CA |
| BB_K32 QPCR R | AAA TTG GTC TCT ACT TCT AAA GC |
| BB_A25 QPCR F | TCG CTA GAA GAC GTT GGA ATA ATA GG |
| BB_A25 QPCR R | TTA GCC GCA AGC AAT CTT TCA |
| DbpA qPCR F | AGA AAC TGG AAG TAG TGG TGA |
| DbpA qPCR R | TTG TCA TCT CTT GTA TTC CCA A |
| OspC qPCR F | GTT AGC GGG AGC TTA TGC AA |
| OspC qPCR R | ACA TTT CTT AGC CGC ATC AAT |

**Supplementary Table 1**. Sequences of oligonucleotides used in this study
