# Supplementary material for: Genome-wide screen identifies novel genes required for Borrelia burgdorferi survival in its Ixodes tick vector
Source: PLoS Pathog. 2019 May 14;15(5):e1007644. doi: 10.1371/journal.ppat.1007644 (PMC6516651; doi:10.1371/journal.ppat.1007644)
Supplement: S2 Table — Rep., replicon; RPKM, Reads Per Kilobase Million; q, False Discovery Rate adjusted p-value; P, parental strain * Repressed by RpoS 20.† Activated by RpoS 19,20,‡ Shading indicates genes with identical or near-identical sequence. (DOCX) [file ppat.1007644.s002.docx]

**Supplementary Table 2.** Genes differentially expressed at least 2-fold in the Tn::*bb0017* mutant compared to the parental strain

| **Rep.** | **Locus** | **Gene name** | **RPKM** | | **log2 (fold change) Tn::*bb0017*/WT** | **q** |
| --- | --- | --- | --- | --- | --- | --- |
|  |  |  | **WT** | **Tn::*bb0017*** |  |  |
| lp5‡ | *bbt06* |  | 66.5 | 3.7 | -4.8 | <2.5E-14 |
| lp21‡ | *bbu11* |  | 66.5 | 3.7 | -4.8 | <2.5E-14 |
| chr | *bb0242** |  | 258.8 | 53.0 | -2.29 | 2.5E-14 |
| lp5 | *bbt04* |  | 100.1 | 21.6 | -2.21 | <2.5E-14 |
| chr | *bb0241** | *glpK* | 3422.8 | 819.3 | -2.06 | <2.5E-14 |
| chr | *bb0240** | *glpF* | 3845.0 | 926.8 | -2.05 | <2.5E-14 |
| chr | *bb0243** | *glpD* | 1932.9 | 570.2 | -1.76 | <2.5E-14 |
| lp28-1 | *bbf20** |  | 133.0 | 43.1 | -1.62 | 4.5E-03 |
| lp54 | *bba62** | *lp6.6* | 335158.0 | 142504.0 | -1.23 | 3.6E-08 |
| cp32-6‡ | *bbm37* |  | 7.8 | 3.5 | -1.14 | 1.9E-02 |
| cp32-7‡ | *bbo38* |  | 7.7 | 3.5 | -1.13 | 2.0E-02 |
| lp28-1 | *bbf32* |  | 5.6 | 2.4 | -1.19 | 6.4E-06 |
| lp54 | *bba23* |  | 449.5 | 216.5 | -1.05 | 3.5E-06 |
| lp28-1 | *bbf19* |  | 18.6 | 9.2 | -1.02 | 1.3E-02 |
| lp54 | *bba11* |  | 69.3 | 34.3 | -1.02 | 9.0E-05 |
| lp21 | *bbu04* |  | 58.8 | 29.2 | -1.01 | 7.6E-05 |
| cp32-6 | *bbm38*† | *ospF* | 73.9 | 148.3 | 1.00 | 2.4E-05 |
| lp36 | *bbk12* |  | 15.7 | 33.9 | 1.11 | 1.8E-04 |
| lp54 | *bba0078* |  | 185.5 | 405.2 | 1.13 | 1.4E-04 |
| lp38 | *bbj24*† |  | 23.3 | 50.9 | 1.13 | 2.7E-05 |
| lp38 | *bbj26* |  | 36.4 | 83.0 | 1.19 | 3.0E-06 |
| lp54 | *bba33*† |  | 5.2 | 11.9 | 1.20 | 2.0E-02 |
| lp36 | *bbk07*† |  | 111.2 | 260.3 | 1.23 | 5.0E-09 |
| lp54 | *bba24*† | *dbpA* | 303.9 | 740.9 | 1.29 | 4.8E-13 |
| lp54 | *bba34*† | *oppA5* | 31.6 | 81.3 | 1.36 | 5.7E-10 |
| cp26 | *bbb19* | *ospC* | 3812.9 | 9888.0 | 1.37 | 1.5E-10 |
| lp38 | *bbj02a* |  | 1.4 | 3.6 | 1.39 | 5.0E-02 |
| chr | *bb0844*† |  | 109.9 | 304.5 | 1.47 | <2.5E-14 |
| lp38 | *bbj23*† |  | 80.8 | 238.0 | 1.56 | 4.4E-14 |
| lp54 | *bba64*† |  | 236.7 | 743.9 | 1.65 | <2.5E-14 |
| lp54 | *bba36*† |  | 319.2 | 1004.1 | 1.65 | <2.5E-14 |
| lp28-1 | *bbf01* |  | 13.0 | 42.8 | 1.71 | 9.1E-12 |
| cp32-6 | *bbm27* |  | 56.6 | 190.7 | 1.75 | 6.1E-14 |
| cp32-1 | *bbp27* |  | 56.4 | 191.1 | 1.76 | 4.4E-14 |
| chr | *bbr02*† |  | 649.3 | 2288.3 | 1.82 | 9.6E-03 |
| lp54 | *bba25*† | *dbpB* | 170.8 | 604.4 | 1.82 | <2.5E-14 |
| lp28-3 | *bbh41* |  | 4.8 | 17.5 | 1.85 | 1.5E-05 |
| lp36 | *bbk32*† | *bbk32* | 122.2 | 441.1 | 1.85 | <2.5E-14 |
| chr | *bbr04* |  | 624.7 | 2273.7 | 1.86 | 8.1E-03 |
| lp54 | *bba37*† |  | 14.2 | 68.2 | 2.27 | <2.5E-14 |
| chr | *bbr05* |  | 628.7 | 4387.4 | 2.80 | 8.5E-06 |

Rep., replicon; RPKM, Reads Per Kilobase Million; q, False Discovery Rate adjusted p-value; P, parental strain

* Repressed by RpoS 20.† Activated by RpoS 19,20,‡ Shading indicates genes with identical or near-identical sequence
