# Supplementary figures and images for: Genome-wide screen identifies novel genes required for Borrelia burgdorferi survival in its Ixodes tick vector
Source: PLoS Pathog. 2019 May 14;15(5):e1007644. doi: 10.1371/journal.ppat.1007644 (PMC6516651; doi:10.1371/journal.ppat.1007644)

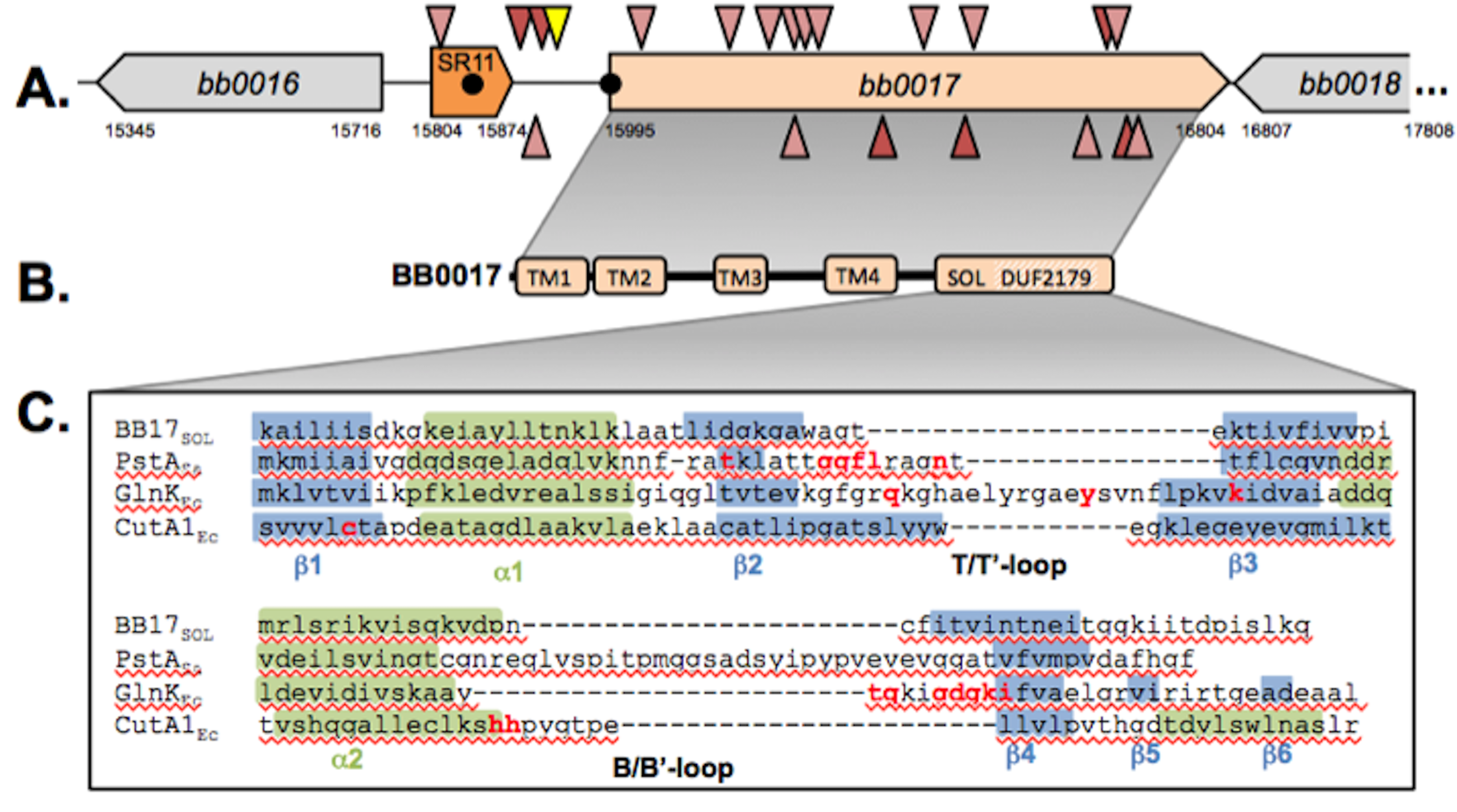

Supplement: S1 Fig — (A) Genomic context of bb0017. Chromosomal coordinates in the B. burgdorferi B31 genome are indicated below the genes. The putative sRNA SR0011 identified in the bb0016-bb0017 intergenic region is shown [69]. Black circles indicate the two annotated bb0017 start sites at positions 15845 and 15995. Triangles indicate the approximate locations of all Tn insertions present in the B. burgdorferi Tn library [43,45]. Triangles located above the gene represent Tn insertions on the positive strand, while triangles located below the gene represent Tn insertions on the reverse strand. Shading indicates the median frequency ratio for a particular Tn mutant following exposure to H2O2 [43]: dark red: frequency ratio < 0.25; light red: frequency ratio <0.5; yellow: frequency ratio <1. (B) Predicted membrane topology of BB0017 [55]. TM, transmembrane domain; SOL, soluble domain. A conserved domain of unknown function (DUF2179) is shown. (C) Amino acid alignment of the soluble domain of BB0017 (BB0017SOL) with the PII-like protein PstA from Staphylococcus aureus (PstASa), the PII protein GlnK from Escherichia coli (GlnKEc), and the PII-like CutA protein from E. coli (CutA1Ec). The predicted secondary structure of BB0017SOL is indicated [56], along with the known secondary structures for PstASa (PDB code 4D3G), GlnKEc (PDB code 1GNK), and CutA1Ec (PDB code 1NAQ) [65]. Beta sheets (blue shading) and alpha helices (green shading) are indicated. Conserved residues important for ligand binding or regulatory function in the different protein families are shown in red type. For GlnKEc, these residues include the highly conserved Gln-39 and Lys-58 residues involved in salt bridge formation, the Tyr-51 site of uridylylation, and the TGxxGDGKI motif involved in ATP binding [58]. For PstASa, these residues include the highly conserved Thr-28, GGFL motif, and Asn- 31 residue involved in c-di-AMP binding [64]. For CutA1Ec, these residues include Cys-16, His-83, and His-84 involved in copper bin [file ppat.1007644.s003.tif]

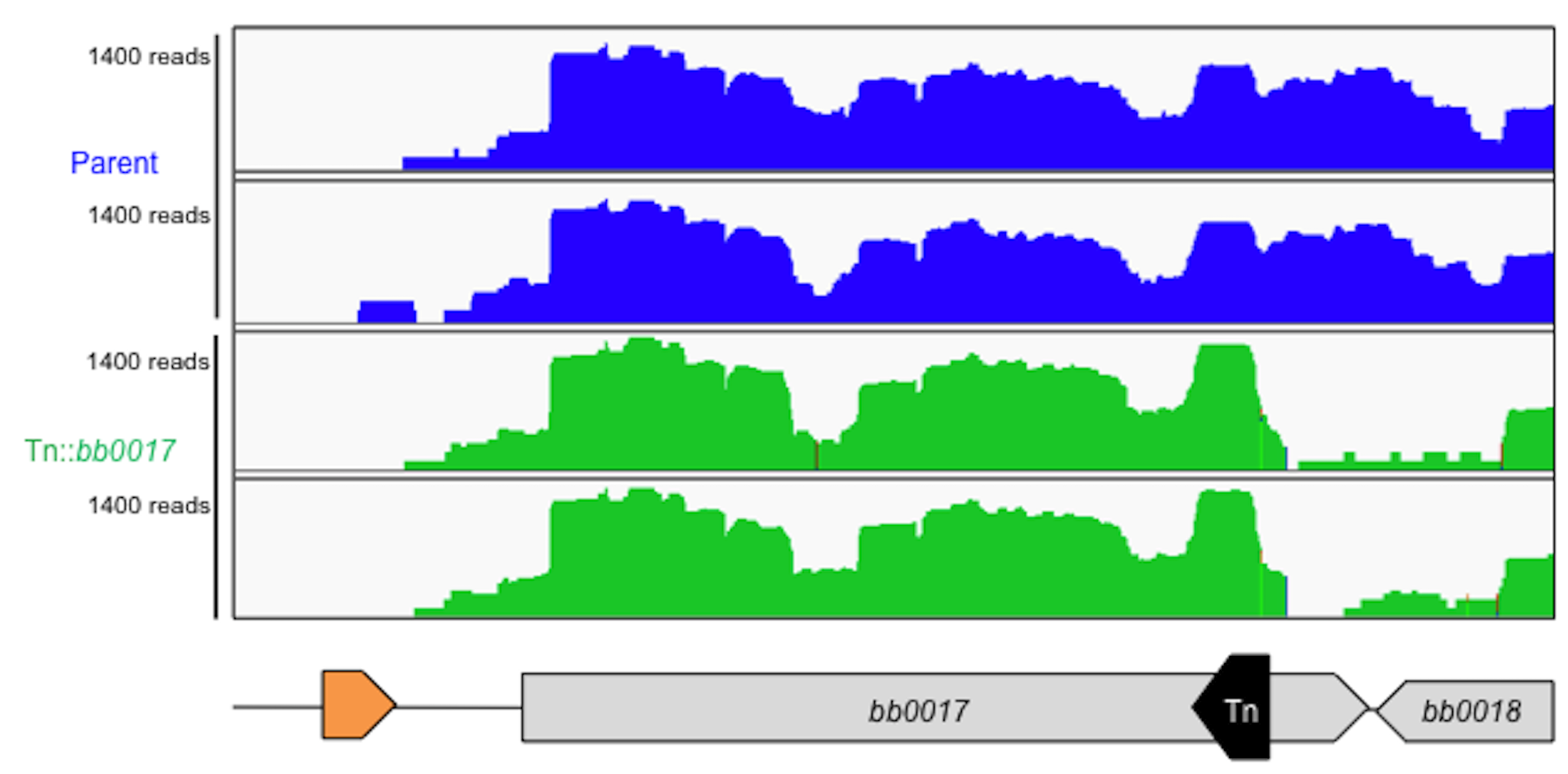

Supplement: S2 Fig — The location of the transposon insertion on the reverse strand is indicated with a triangle. The red/green lines in the Tn::bb0017 mutant represent SNPs in the RNA-seq reads relative to the genome sequence. (A= green, C =blue, G yellow, T red). (TIF) [file ppat.1007644.s004.tif]

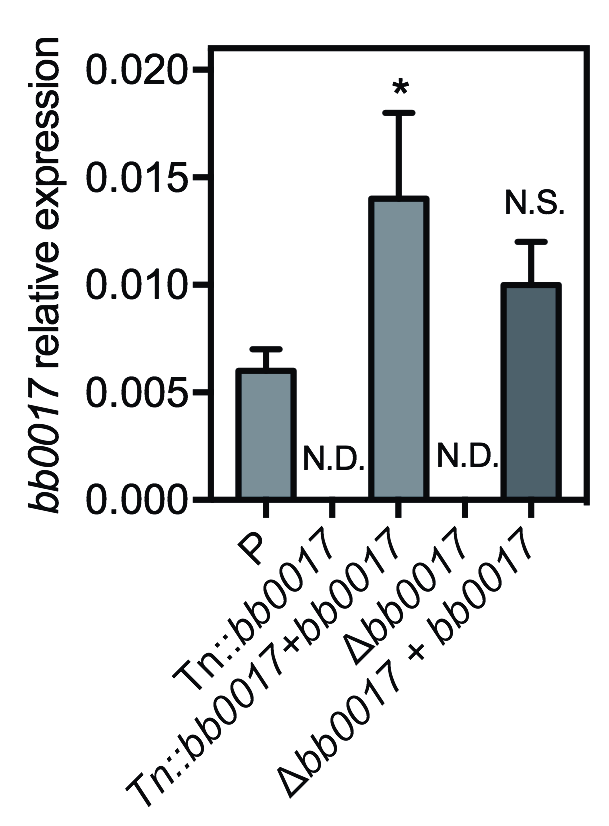

Supplement: S3 Fig — qRT-PCR for bb0017 expression in the parental strain 5A18NP1 (P), the Tn::bb0017 mutant, a bb0017 deletion mutant (bb0017), and strains in which expression of or bb0017 was restored in the bb0017 mutant backgrounds. Expression of bb0017 was normalized to expression of the B. burgdorferi housekeeping gene flaB using the ΔCT method. (TIF) [file ppat.1007644.s005.tif]
